# Post-translational modification networks as master regulators of influenza virus replication, host adaptation, and immune evasion

**DOI:** 10.3389/fimmu.2026.1816377

**Published:** 2026-06-18

**Authors:** Chengxun Jin, Bingya Zhang, Yihang Gao

**Affiliations:** 1 Department of Otolaryngology, The Second Hospital of Jilin University, Changchun, China; 2 Department of Ultrasound Medicine, The Second Hospital of Jilin University, Changchun, China; 3 Department of Laboratory Medicine, The Second Hospital of Jilin University, Changchun, China

**Keywords:** immune evasion, influenza viruses, metabolic reprogramming, post-translational modifications, vaccine strategies

## Abstract

Influenza viruses remain major global health threats due to their rapid evolution and complex interactions with host regulatory systems. While genetic variation is a key driver of viral pathogenicity and host adaptation, growing evidence indicates that post-translational modifications (PTMs) provide an additional regulatory layer that can influence viral replication, immune evasion, and metabolic remodeling. In this review, we synthesize current knowledge on how coordinated PTM networks, including phosphorylation, ubiquitination, SUMOylation, glycosylation, acetylation, lipidation, and RNA methylation, are implicated across multiple stages of the influenza virus life cycle. Accumulating studies indicate that phosphorylation and ubiquitin signaling can fine-tune polymerase activity and ribonucleoprotein trafficking, whereas SUMOylation and acetylation modulate polymerase function and host immune antagonism. Glycosylation remodeling of viral glycoproteins is closely associated with antigenic evolution and immune escape, while epitranscriptomic RNA methylation and PTM-linked metabolic pathways reshape host environments in ways that may support replication. Notably, many mechanistic insights derive from cell-based or model organism systems, and the relative contribution of specific PTM events during human infection remains incompletely defined. A systems-level view of PTM networks highlights emerging regulatory vulnerabilities at the host–virus interface and supports further evaluation of PTM-modulating enzymes as potential targets for host-directed antiviral and vaccine strategies.

## 1 Introduction

Influenza viruses remain major global health concerns, causing substantial morbidity and mortality in humans and animals and imposing a significant burden on public health (1, 2). Influenza is an acute viral respiratory infection capable of causing seasonal epidemics as well as occasional pandemics (3, 4). This virus is highly contagious and infects several animals, including humans, pigs, and birds. It is a negative-sense, segmented RNA virus belonging to the Orthomyxoviridae family. It has several types: A, B, C, and D, based on their genetic makeup, antigenicity, and host range (5). Although substantial effort has focused on defining the genetic basis of influenza virus evolution (6–8), accumulating evidence suggests that viral fitness is also shaped by post-translational modifications (PTMs) at the host–virus interface (6–8). These reversible PTMs, including phosphorylation, ubiquitination, SUMOylation, glycosylation, acetylation, methylation, and ubiquitin-like modifications, act as dynamic regulators of viral replication, immune evasion, and host cellular reprogramming (8–15).

PPTMs have been shown to modulate multiple aspects of the influenza virus life cycle (16–18). Key steps of the viral life cycle, including polymerase function, ribonucleoprotein transport, virion assembly, and genome replication, are influenced by site-specific phosphorylation and ubiquitin-related PTMs. In parallel, SUMOylation and acetylation of the influenza polymerase can modulate its structural configuration, transcriptional capacity, and immune antagonistic functions (19–23). Concurrently, glycosylation of influenza surface glycoproteins modulates antigenicity and receptor binding, thereby contributing to immune evasion and host adaptation (24–27). Influenza PTM networks are closely interconnected with host PTM machinery, as viruses engage components of the host kinome, ubiquitinome, and metabolome to establish cellular environments that favor replication (15, 28, 29).

Beyond direct modification of viral proteins, PTM-mediated signaling also reshapes host immune and metabolic pathways. This indirectly affects the outcome of infection at the organism level (15, 30). Kinase-driven phosphorylation cascades, ubiquitin-mediated innate immune responses, and epitranscriptomic RNA modifications collectively influence the balance between antiviral restriction and viral propagation (31–33). The two-way nature of these interactions, in which the host uses PTMs for antiviral defense and viruses exploit them for propagation, underscores the importance of PTM-mediated signaling in determining pathogenicity and host range (34–38).

In this Review, we synthesize emerging evidence to position PTMs as an integrated regulatory layer in influenza virus biology. We discuss how integrated PTM systems regulate the viral replication machinery, reprogram the host signaling landscape, facilitate antigenic variation, and create targets for host-directed approaches to viral infection. A systems-level view of PTM biology may help bridge viral regulation and evolution and inform the development of host-directed therapeutics and next-generation vaccines. In this review, we focus on how interconnected PTM networks contribute to the regulation of viral replication, immune modulation, and metabolic rewiring during influenza infection.

## 2 Integrated PTM networks at the host–influenza interface

PTM networks at the host–influenza interface involve multi-layered cross-talk that influences viral replication, immune evasion, and cell-fate decisions during infection. While multiple mechanisms have been proposed across diverse models, their relative contributions can differ across strains and host systems, highlighting the need for integrative and quantitative validation. For example, phosphorylation-dependent ubiquitination has been reported in the interaction between NS1 and DNMT3B, which promotes cytoplasmic relocalization of the complex and facilitates K48-linked polyubiquitination with downstream epigenetic effects (39). An example of the SUMO-ubiquitin switch is the influenza virus-dependent SUMO pathway that triggers the activation of endogenous retroviruses to enhance the host’s antiviral immune response (40). PTM-dependent regulation of cell-death gating also involves TAK1 activation. During influenza infection, TAK1 signaling has been shown to suppress RIPK3-mediated apoptosis and RIPK1-dependent necroptosis (41). In addition, TRIF–TAK1 signaling has been reported to restrain activation of the caspase-8/3–GSDMD/E axis, thereby limiting pyroptotic responses in influenza A virus–infected airway epithelial cells (42). Epigenetic PTM layers, including H3K79 methylation, have also been implicated in interferon-mediated antiviral responses during influenza infection (43). DNA methylation pathways have likewise been linked to modulation of innate immune responses following influenza vaccination (44). The following sections examine the reported roles of viral and host PTMs in replication, immune modulation, and host adaptation.

## 3 Phosphorylation-dependent regulation of viral replication machinery

### 3.1 Polymerase phosphorylation (PB1, PB2, PA)

Phosphorylation of influenza A virus (IAV) polymerase subunits represents an important regulatory mechanism that can modulate polymerase activity, ribonucleoprotein (vRNP) assembly, and viral fitness. Site-specific phosphorylation events can exert either activating or inhibitory effects, suggesting that balanced phosphorylation states are important for efficient transcription–replication switching. Phosphorylation of the PA subunit at serine 225 has been reported to act as an activating modification that enhances viral replication and virulence. Phosphorylation-deficient mutants show reduced polymerase activity, impaired vRNP assembly, and diminished nuclear accumulation of PA, changes that are associated with attenuated replication in avian and mammalian cells and reduced pathogenicity *in vivo* (19). In contrast, phosphorylation at other PA residues may impair polymerase activity. Phosphorylation at tyrosine 393 has been shown to disrupt interactions between the polymerase complex and the viral RNA 5′ termini, thereby impairing promoter binding and viral RNA synthesis. These observations support a model in which phosphorylation can function as a structural switch influencing polymerase configuration (45). A comparable inhibitory regulation mechanism exists in the PB2 subunit. Phosphorylation at serine 181 has been reported to diminish PB2 stability and nuclear localization, changes that correlate with decreased polymerase activity, impaired vRNP synthesis, and reduced viral replication and pathogenicity (46). These findings indicate that distinct phosphorylation sites across polymerase subunits can exert opposing functional effects, collectively contributing to the fine regulation of transcriptional competence. Together, these phosphorylation events (e.g., activating PA S225 and inhibitory PA Y393 and PB2 S181) illustrate a multilayered regulatory mechanism that modulates polymerase dynamics during influenza virus replication.

### 3.2 NP phosphorylation and genome replication

Phosphorylation of the IAV nucleoprotein (NP) is also a key PTM that modulates viral genome replication, polymerase activity, and pathogenicity by affecting NP oligomerization, nuclear transport, and RNP formation. Mass spectrometry revealed NP S69 as a new PTM site highly conserved across different IAV subtypes (20). The dephosphorylation-mimetic mutation (S69A) had a negligible effect on replication in MDCK cells and on virulence in mice, maintaining lung titers and disease (20). On the other hand, constitutive phosphorylation mimicking S69E completely inhibits viral viability, affecting polymerase and RNP formation (20). S69 phosphorylation interferes with NP-PB2 interactions, which are critical for heterotrimeric polymerase reconfiguration. It inhibits NP import into the nucleus by reducing NP-importin-α3 interactions, as evidenced by reduced nuclear fluorescence in transfected cells (20). Polo-like kinase 3 (PLK3) phosphorylates NP at S482 and appears to be important for swine IAV replication. PLK3 overexpression, which occurs after infection, interacts with NP and promotes S482 phosphorylation and NP oligomerization, as revealed by co-immunoprecipitation and mass spectrometry (47). This enhances viral polymerase activity and RNP formation, resulting in increased titers of progeny viruses in NPTr cells (47). The S482A mutation eliminates PLK3 enhancement, resulting in reduced replication and oligomerization without affecting NP stability or localization (47). PLK3 knockdown or inhibitor (BI-2536) inhibits NP phosphorylation, polymerase activity, and viral multiplication, supporting a proviral role for PLK3 (47). Overall, phosphorylation of NP at S69 and S482 appears to exert opposing effects on genome replication. Phosphorylation at S69 is largely restrictive, as it disrupts polymerase interactions and nuclear import, whereas phosphorylation at S482 is generally facilitative by promoting NP oligomerization. Much of the mechanistic evidence has been obtained from cell-based assays and targeted mutagenesis, and the quantitative contribution of each site to viral fitness may vary across strains and host contexts.

### 3.3 M1 and NEP phosphorylation and nuclear trafficking

Phosphorylation of IAV matrix protein 1 (M1) and nuclear export protein (NEP) is involved in vRNP nuclear export, multimerization, and replication through site-specific PTMs, affecting protein interactions and subcellular localization. S23C/S24L/S25L mutations, which abolish phosphorylation, affect vRNP export and reduce infectivity and virulence in mice, as supported by/consistent with lower lung titers and pathology (48). ATM/CK2 kinases phosphorylate this motif, and inhibitors (KU-55933/CAY10561) or knockouts prevent NEP-kinase binding, vRNP export, and replication (48). Competitive NEP peptide mimics impair this connection, inhibiting replication in both cells and animals (48). Phosphorylation of Y132 of IAV M1 is involved in nuclear import. Y132 is identified as a target through mass spectrometry and is crucial for binding with importin α1. Y132F/A mutations, which mimic dephosphorylation, abolish binding with importin α1 and affect nuclear import and replication (49). Janus kinase inhibition prevents Y132 phosphorylation, supporting a role for this modification in M1 trafficking and viral assembly (49).

M1 T108 phosphorylation governs multimerization and STRIPAK complex association (50). T108 phosphorylation, conserved across IAV strains, promotes M1 self-association at the membrane, preventing premature nuclear entry (50). T108A (dephosphorylation mimic) results in increased nuclear entry but affects vRNP export, while T108D (phospho-mimetic) results in cytoplasmic retention (50). IAV infection redistributes STRIPAK subunits (STRN/STRN3) to cytosolic/perinuclear clusters that colocalize with M1; STRIPAK inactivation (siRNA/inhibitors) inhibits M1 polymerization and replication (3). Collectively, phosphorylation events at M1 Y132/T108 and the NEP serine cluster contribute to the spatiotemporal regulation of M1/NEP activities and vRNP trafficking. Kinase inhibitors targeting these events have shown antiviral potential in experimental models.

### 3.4 Kinase recruitment strategies by influenza viruses

Influenza viruses exploit host kinase signaling networks extensively to coordinate viral transcription, vRNP trafficking, and cellular conditions favorable to replication. Rather than depending solely on constitutive phosphorylation, viral proteins actively drive kinase activity via signaling mimicry, scaffolded kinase assembly, and selective interactions with kinase-regulated host enzymes. The multifunctional nonstructural protein NS1 binds to the regulatory p85β subunit of phosphoinositide 3-kinase (PI3K), causing isoform-specific redistribution and activation of PI3K complexes that promote viral fitness. This mimics the mechanism of oncogenic kinase activation (51). NS1 function is further influenced by host kinase activity later in infection: phosphorylation at serine 205, mediated in part by casein kinase 2 (CK2), improves interaction with the cellular factor DDX21 and promotes viral polymerase activity, emphasizing phosphorylation-dependent recruitment of host cofactors that optimize transcriptional output (28). Influenza infection also stimulates the formation of spatially structured kinase assemblies that control vRNP trafficking. The PKCα-MEK1-ERK2 signaling module phosphorylates viral NP, facilitating nuclear export of vRNPs and productive virus replication. PKCα acts as a scaffold to sustain the multikinase complex (52).

In addition to direct interactions with viral proteins, influenza viruses also manipulate kinase-regulated host factors that play important roles in viral RNA synthesis. The CK2 kinase-mediated phosphorylation of the cap methyltransferase CMTR1 enhances its recruitment to RNA polymerase II and, as such, enhances co-transcriptional RNA cap methylation reactions that are subsequently utilized in cap snatching-dependent viral transcription (53). Kinase rewiring occurs in the early stages of infection, when receptor activation triggers Cdc42-dependent signaling pathways that increase filopodia formation and viral uptake, suggesting that kinase-driven cytoskeletal remodeling improves entrance efficiency (54). Influenza viruses employ multiple kinase recruitment strategies, including signaling mimicry, scaffolded kinase cascades, and modulation of phosphorylated host enzymes, to regulate different steps of the replication cycle (**Table 1**). The relative impact of specific kinase modules is often context-dependent, and further *in vivo* validation is required.

**Table 1:** Post-translational modifications of viral and host proteins regulate influenza virus replication by modulating polymerase activity, viral entry, immune signaling, and host metabolic pathways.

PTM type	Protein target	Modified residue(s)	Mechanistic effect	Impact on viral infection	Ref.
Phosphorylation	PA	S225	Enhances polymerase activity, vRNP assembly, and nuclear accumulation	Increases replication and virulence	(19)
Phosphorylation	PA	Y393	Prevents 5′ RNA promoter binding	Blocks polymerase function	(45)
Phosphorylation	PB2	S181	Reduces PB2 stability and polymerase activity	Restricts replication	(46)
Phosphorylation	NP	S69	Disrupts NP–PB2 interaction and nuclear import	Reduces replication	(20)
Phosphorylation	NP	S482	Promotes NP oligomerization and vRNP assembly	Enhances replication	(47)
Phosphorylation	NEP	S23/S24/S25	Required for vRNP nuclear export	Promotes infectivity	(48)
Phosphorylation	M1	Y132	Controls importin−α binding and nuclear import	Essential for replication	(49)
Phosphorylation	M1	T108	Regulates multimerization and STRIPAK binding	Controls replication	(50)
Phosphorylation	NS1	PI3K pathway	Activates PI3K signaling	Enhances viral fitness	(51)
Phosphorylation	NP	Ser/Thr residues	PKCα–MEK1–ERK2 complex phosphorylates NP	Promotes vRNP export	(28)
Phosphorylation	CMTR1 (host)	P−patch	Enhances RNA cap methylation	Promotes infection	(52)
Phosphorylation	NS1	S205	Enhances NS1−DDX21 interaction	Increases polymerase activity	(53)
Phosphorylation	Cortactin (host)	Multiple	Limits virus entry via cytoskeleton remodeling	Restricts infection	(54)
Ubiquitination	HA	PolyUb	Targets HA for degradation	Restricts infection	(55)
Ubiquitination	NA	K242	Autophagic degradation of NA	Restricts replication	(56)
Ubiquitination	M1	K102	Promotes budding and particle release	Enhances replication	(21)
Ubiquitination	M2	K78	Lysosomal degradation of M2	Restricts infection	(57)
Ubiquitination	M1	K242	Proteasomal degradation via TRIM21	Restricts infection	(58)
Ubiquitination	NP	K351	Disrupts vRNP formation	Restricts replication	(59)
Ubiquitination	PB2	PolyUb	TRIM35-mediated degradation	Restricts infection	(60)
Ubiquitination	NP	PolyUb	TRIM41-mediated degradation	Restricts infection	(35)
Ubiquitination	PB1	K578	Controls polymerase dimerization and RNA synthesis	Regulates replication	(16)
Ubiquitination	AMPK (host)	PolyUb	CRBN degrades AMPK, altering metabolism	Promotes replication	(61)
Ubiquitination	Polymerase complex	Multiple	Enhances polymerase activity independent of degradation	Promotes replication	(62)
Ubiquitination	PB2	PolyUb	CRL4-mediated non−degradative ubiquitination	Promotes infection	(29)
Ubiquitination	Paxillin δ (host)	K68	Regulates endosomal entry	Promotes infection	(63)
Ubiquitination	HA (host control)	PolyUb	MARCH10 expression modulates HA levels	Restricts infection	(64)
Ubiquitination	PB2	K29-linked chains	Nonproteolytic ubiquitination is required for production	Promotes infection	(36)
SUMOylation	NP	K7/K48/K77/K113	Enhances NP function	Promotes replication	(65)
SUMOylation	NP	K7/K48/K87	Enhances NP SUMOylation	Promotes replication	(66)
SUMOylation	NP	K4/K7	Controls intracellular trafficking	Essential for growth	(17)
SUMOylation	PB1	K612	Maintains RNA−binding capacity	Essential for replication	(22)
SUMOylation	NS1	K131	Enhances replication capacity	Promotes replication	(67)
SUMOylation	M1	A215	Maintains stability and nuclear export	Promotes virulence	(68)
SUMOylation	ANP32A/B (host)	Multiple	Facilitates NS2−dependent polymerase adaptation	Promotes replication	(38)
SUMO interaction	NS2	SIM motif	Enhances vRNP assembly	Promotes adaptation	(69)
SUMO switch	TRIM28 (host)	SUMO loss	Induces antiviral immunity	Restricts infection	(40)

## 4 Ubiquitination and deubiquitination networks regulating viral protein stability

### 4.1 Viral protein ubiquitination

Ubiquitination of IAV proteins represents a dynamic post-translational regulatory mechanism in which host E3 ligases can mediate both antiviral restriction and proviral regulatory activities through linkage-specific ubiquitin signaling. Distinct ubiquitin chain topologies, including degradative K48-linked modifications and non-proteolytic K63 or short-chain ubiquitination, have been reported to regulate viral protein stability, vRNP assembly, and virion morphogenesis. Multiple intrinsic restriction mechanisms have been shown to target viral surface glycoproteins for ubiquitin-mediated degradation. The E3 ligase MARCH10 has been reported to polyubiquitinate HA, thereby promoting its degradation, reducing viral infectivity, and enhancing interferon-associated host defense responses (55). Similarly, the host factor HSPA1L binds neuraminidase (NA) and promotes ubiquitination at K242, which is associated with NBR1-dependent autophagic degradation and reduced viral release. Mutation of this site confers partial resistance to host restriction (56). Ubiquitination-dependent regulation also extends to the ion channel M2, where MARCH 8 catalyzes K63-linked polyubiquitination at K78, guiding M2 to lysosomal degradation and inhibiting virion budding (21).

Similarly, the internal viral proteins are controlled by ubiquitin signaling pathways. TRIM21 facilitates K48 ubiquitination of the matrix protein M1 at K242, triggering its degradation and inhibiting viral replication (58). TRIM41 and Pirh2 recognize and ubiquitinate the viral NP via distinct ubiquitination pathways. TRIM41 triggers NP degradation by polyubiquitination (57), and Pirh2 triggers short-chain ubiquitination of K351 of NP and inhibits NP-PB2 interaction and vRNP formation without inducing degradation (59). TRIM35 exhibits dual antiviral roles by triggering K63 ubiquitination of TRAF3 and K48 ubiquitination of the viral polymerase subunit PB2, and by inducing PB2 degradation (60). Some ubiquitination processes facilitate viral replication by triggering K63 ubiquitination of M1 at K102 by the proteasome regulatory component PSMD12, thereby inducing the formation of virus-like particles and efficient virion budding (35). In addition, proteome-wide studies identified many ubiquitination sites on polymerase proteins and revealed ubiquitination of PB1 K578, with implications for polymerase dimerization and vRNA synthesis (16). Collectively, these findings indicate that influenza virus infection is shaped by a bidirectional ubiquitin regulatory network. The balance between antiviral and proviral ubiquitin signaling may contribute to replication efficiency and host adaptation.

### 4.2 Proviral *vs* antiviral ubiquitin signaling

Ubiquitin signaling during IAV infection exerts both proviral and antiviral effects through substrate-specific and linkage-dependent modifications that regulate host metabolism, polymerase activity, viral entry, and epithelial defense mechanisms. The functional outcome of ubiquitination is therefore largely influenced by the identity of the modified protein and the cellular pathways engaged during infection. Several ubiquitin pathways have been reported to promote viral replication by reprogramming host metabolic and replication environments. The CRL4 substrate adaptor cereblon (CRBN) enhances IAV and IBV replication by catalyzing proteasomal degradation of AMP-activated protein kinase (AMPK), shifting cellular metabolism toward anabolic lipid synthesis and lipid-droplet accumulation that favors viral propagation; genetic or pharmacological inhibition of CRBN stabilizes AMPK and suppresses viral replication *in vitro* and *in vivo* (61). Ubiquitination also directly enhances polymerase activity: ubiquitination of polymerase subunits increases viral RNA synthesis independently of ribonucleoprotein assembly, indicating a non-degradative regulatory role of ubiquitin signaling in transcriptional activation (62).

**Figure 1:**
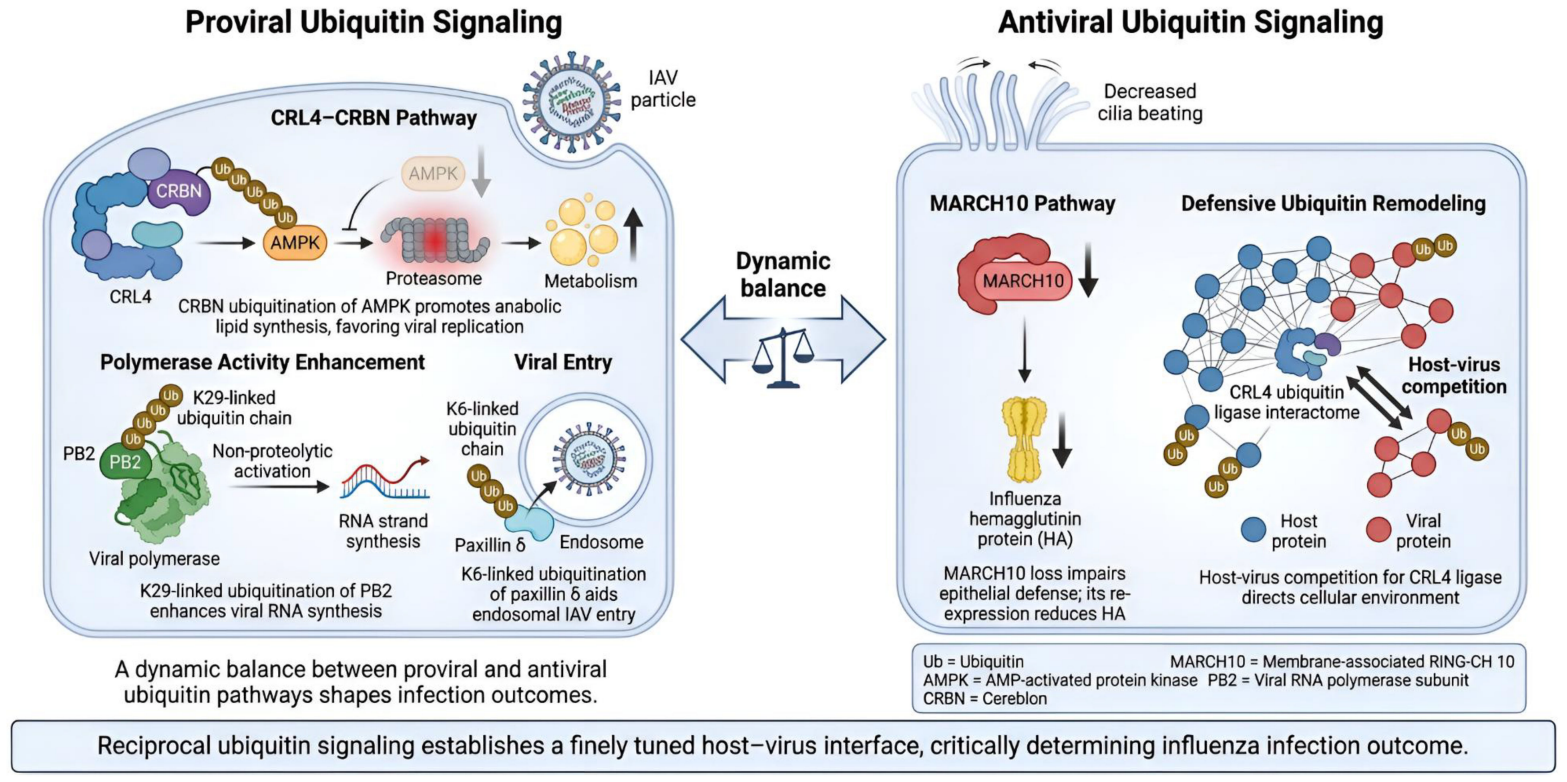
Proviral versus antiviral ubiquitin signaling during influenza virus infection. Ubiquitin pathways promote influenza replication through CRL4–CRBN-mediated AMPK degradation, metabolic reprogramming, PB2/polymerase ubiquitination, and paxillin δ-dependent endosomal viral entry. In contrast, antiviral ubiquitin signaling, including MARCH10-mediated epithelial defense and infection-induced CRL4 interactome remodeling, shapes host–virus competition and determines infection outcome.

Consistently, cullin 4-based E3 ligases mediate non-proteolytic ubiquitination of the PB2 polymerase subunit, which includes K29-linked chains that are necessary for efficient virion production and optimal progression of the viral life cycle (29, 36). Ubiquitination of host trafficking factors is also important in the early stages of infection. K6-linked ubiquitination of paxillin δ is important for efficient infection, as it regulates endosomal-dependent viral entry (63). In contrast, antiviral ubiquitin pathways mediate epithelial defense functions. Influenza infection leads to downregulation of the E3 ligase MARCH10, which is important for airway epithelial cell defense. The downregulation of MARCH10 leads to decreased ciliary beat frequencies, while re-expression of MARCH10 leads to decreased levels of influenza hemagglutinin, indicating a role in epithelial defense (64). Infection-induced remodeling of CRL4 ubiquitin ligase interactomes is an example of the competition between the host and the influenza virus for control of the ubiquitin pathway, which is important in generating a cellular environment that is either conducive to the influenza life cycle or inhibits it (29). Collectively, these studies indicate that influenza virus infection is shaped by a dynamic balance between proviral and antiviral ubiquitin signaling pathways. This reciprocal regulation influences infection outcomes (**Figure 1**; **Table 1**).

## 5 SUMOylation in intracellular trafficking, adaptation, and virulence

### 5.1 NP, PB1, NS1 SUMOylation

SUMOylation has emerged as an important regulatory mechanism influencing IAV vRNP assembly, trafficking, and replication. Multiple studies indicate that NP, the polymerase subunit PB1, and accessory non-structural proteins can be directly targeted by the host SUMO conjugation machinery, revealing a coordinated post-translational regulatory layer that optimizes viral fitness. NP is among the most extensively reported SUMO-modified IAV proteins. Early mechanistic analyses established that NP is SUMOylated at conserved N-terminal lysines, particularly K7. That Loss of these modifications has been associated with premature cytoplasmic localization, impaired intracellular trafficking, and marked attenuation of viral growth, highlighting SUMOylation as essential for productive infection (17). Subsequent host-factor investigations revealed that the SUMO E3 ligase PIAS2 directly interacts with NP, promoting NP SUMOylation and thereby increasing avian influenza virus replication in both chicken and duck systems (65, 66). Mutational mapping revealed many NP SUMO acceptor residues, including K7, K48, K77, K87, and K113, demonstrating that multi-site SUMOylation stabilizes functional NP conformations necessary for successful vRNP assembly (65, 66). Notably, the deSUMOylating enzyme SENP1 counteracts NP SUMOylation and has been reported to suppress viral replication, indicating that dynamic SUMOylation is essential for efficient viral infection (66).

Beyond NP, SUMOylation also regulates the enzymatic core of the viral polymerase. A conserved SUMOylation site at PB1 K612 appears to be functionally important for polymerase activity, as it maintains PB1 binding to viral RNA without affecting protein stability or localization (22). Viruses carrying PB1 SUMOylation-deficient mutations exhibit markedly reduced replication, attenuated virulence in mice, and impaired airborne transmission in ferrets, underscoring the direct contribution of PB1 SUMOylation to pathogenesis and host-to-host spread (22). These findings support a model in which SUMOylation functions as a structural regulator of polymerase activity rather than merely a stability determinant.

SUMO modification also affects the non-structural protein NS1, which modulates host antiviral responses. A SUMO acceptor site at NS1 K131 has been reported to promote viral replication; SUMOylation-deficient NS1 mutants exhibit reduced viral growth, supporting a role for NS1 SUMOylation in immune evasion and replication efficiency (67). These findings, together with evidence that other non-degradative PTMs, such as ubiquitination, regulate polymerase components (36), suggest that SUMOylation is part of a broader PTM network coordinating polymerase complex activity. Collectively, these data indicate that host SUMO ligases can modify NP, PB1, and NS1, thereby contributing to vRNP trafficking, RNA synthesis, and immune antagonism. Disruption of individual SUMOylation events often attenuates viral replication in experimental systems.

### 5.2 SUMO-dependent host adaptation

Regulatory networks mediated by SUMO are important for IAV host adaptation by regulating viral protein function and the utilization of species-specific host factors. Recent studies suggest that not only does SUMOylation play an important role in viral replication, but it also facilitates cross-species transmission through dynamic interactions between viral proteins and the host SUMOylation machinery. In terms of virion structure and stability, SUMOylation of the influenza virus matrix protein M1 plays an important role in the adaptation of highly pathogenic influenza A virus subtype H5N1 strains in mammals. The SUMOylation of M1 at residue A215 stabilizes the protein and is important for its optimal nuclear export in complex with vRNP, thereby increasing viral replication and pathogenicity in mammals. Loss of SUMOylation results in significantly attenuated viral growth and pathogenicity (68). These findings suggest that SUMO-dependent structural optimization of viral proteins may contribute to host-specific fitness.

Virus-protein interactions with species-restricted cofactors also influence host adaptation through SUMO-regulated interactions. Avian influenza viruses usually replicate inefficiently in mammalian cells because of suboptimal interactions between avian polymerases and human ANP32A/B. PIAS2α-mediated SUMOylation of human ANP32A/B generates SUMO peptides that are specifically recognized by a conserved SIM domain of the nuclear export protein NEP formerly referred to as NS2, thereby recruiting ANP32A/B to vRNPs and increasing polymerase activity in mammalian cells (38). Indeed, mutations in the NEP SIM compromise vRNP assembly, replication, and pathogenicity in mammalian but not avian cells, suggesting that NEP–ANP32 interactions mediated through SUMOylation may act as a molecular bridge for cross-species polymerase compatibility (69).

Beyond proviral pathways, host SUMO modification adds to antiviral defenses that influence adaptive selection. Influenza infection causes an SUMO flip defined by the loss of SUMO-modified TRIM28, which deactivates endogenous retroviral elements and generates immunostimulatory double-stranded RNA that activates interferon signaling pathways (40). Viral antagonism of this response, including NS1-mediated dsRNA sequestration, demonstrates the evolutionary balance between SUMO-driven host limitation and viral counter-adaptation. Overall, available evidence supports a unified model in which SUMOylation may influence host adaptation through two complementary mechanisms: enabling viral protein function and species-specific cofactor recruitment, while also altering innate immunological constraints that drive adaptive viral evolution (**Table 1**).

## 6 Glycosylation shaping antigenicity, receptor binding, and viral evolution

### 6.1 HA and NA glycosylation dynamics

Glycosylation of the IAV surface glycoproteins HA and NA represents a highly dynamic evolutionary feature that can influence receptor binding, immune escape, virion assembly, and cross-species adaptation. Temporal acquisition, loss, and variation in occupancy of N-linked glycosylation sites collectively contribute to viral fitness by balancing structural stability with antigenic accessibility. Glycans near the receptor-binding site (RBS) of HA can directly influence receptor specificity and host tropism In influenza B viruses, a lineage-defining glycosylation at HA residue 196 governs the breadth of receptor engagement; deletion of this modification increases receptor-binding capacity while modifying lineage prevalence (24). Similarly, glycosylation motifs around residues 158–160 in H3 and H5 viruses have been reported to influence receptor accessibility and antigenicity; acquiring glycan structures at these sites can reduce receptor binding or antibody recognition, while removing them can increase receptor engagement and transmission potential (70, 71). Moreover, glycans within HA have been shown to modulate inter-trimer interactions, as evidenced by the N158 glycan modification in H5 viruses, which alters the higher-order structure of the HA (72). Additionally, glycosylation modifications within antigenic epitopes, including residues 140, 156, and 170, have been shown to control inter-branch antigenic variability, which is associated with vaccine mismatch and evolutionary diversification (73).

Glycan occupancy dynamics may provide an additional layer of regulation. For example, low-occupancy HA glycosylation sites, such as N45 and N144, may influence antibody responses without compromising protein function, suggesting a potential mechanism for fine-tuning antigenic exposure during viral evolution (74). In addition, the emergence of novel glycosylation motifs may enhance structural stability. For example, the introduction of a novel glycosylation site in H5N6 influenza viruses has been reported to stabilize the HA trimer, lower the fusion pH threshold, and facilitate mammalian adaptation (27). NA glycosylation has also been implicated in virus assembly and NA protein stability. Glycosylation of the stalk domains of the NA protein is important for the correct assembly of the NA tetramer and for compensating for local hydrophobicity to promote viral growth (75).

**Figure 2:**
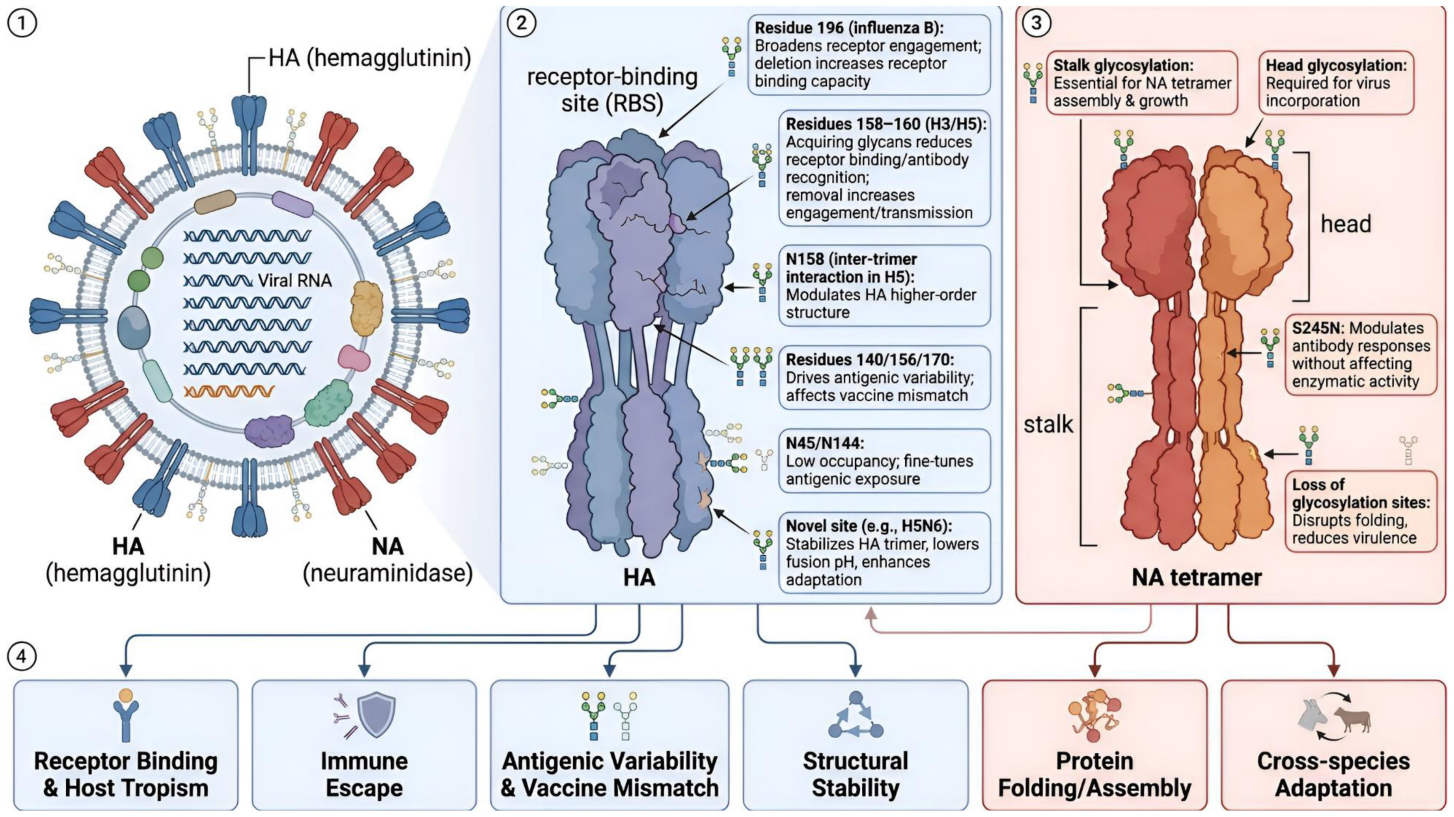
HA and NA glycosylation dynamics in influenza A virus evolution. Dynamic acquisition, loss, and differential occupancy of N-linked glycans on HA regulate receptor-binding-site accessibility, antigenic exposure, HA trimer stability, and host adaptation. NA glycosylation in the stalk and head domains supports tetramer assembly, virion incorporation, antibody-response modulation, protein folding, and viral fitness.

In contrast, NA head glycosylation is important for the correct incorporation of the NA protein into the virus (76). The acquisition of specific NA glycosylation sites has also been demonstrated to play a role in the modulation of antibody responses to the NA protein; for example, the acquisition of an S245N modification at the NA enzyme site may modulate antibody responses to the NA protein without affecting the overall function of the enzyme (77). In addition, the loss of key NA glycosylation sites has been shown to affect protein folding and reduce the virus’s virulence (78). The dynamic remodeling of HA and NA proteins through the acquisition of new glycosylation motifs may thus serve as a combined evolutionary mechanism to modulate receptor binding activity, antibody responses, protein stability, and virus assembly (**Figure 2**; **Table 2**).

**Table 2:** Glycosylation of influenza viral and host proteins regulates receptor binding, antigenicity, and replication outcomes.

Protein	Glycosylation site(s)	Mechanistic function	Biological outcome	Ref.
HA	N196	Controls receptor-binding specificity via RBS glycan presence	Determines lineage-specific tropism	(24)
HA	N45, N144 (low occupancy)	Reduces antibody recognition and receptor binding	Facilitates immune evasion	(74)
HA	N158	Mediates HA–HA glycan interactions, forming filament structures	Modulates receptor engagement	(72)
NA	Stalk glycans	Compensate for hydrophobicity to stabilize the tetramer	Maintains viral growth	(75)
HA	N140, N156, N170	It modulates the antigenic epitopes of the HA head	Drives antigenic variability	(73)
NA	N219	Promotes NA budding and ER export	Enhances replication and virulence	(78)
HA	N158	Enhances VLP assembly and replication	Increases pathogenicity	(70)
HA	N129	Stabilizes the HA trimer and fusion pH	Promotes mammalian adaptation	(27)
HA	N158–160 motif	Blocks RBC receptor binding and alters antigenicity	Modulates immune recognition	(71)
NA	Head glycans	Promote virion incorporation and NA stability	Required for efficient replication	(76)
NA	S245N glycosylation	Modulates antibody binding near the active site	Alters antibody inhibition sensitivity	(77)
HA/NA	Reduced occupancy	Modifies viral glycome to evade immunity	Reduces antibody responses	(26)
HA	Loss at 158/169	Induces ER stress and inflammatory signaling	Enhances virulence	(79)
HA	Introduced glycans	Sterically block antibody binding	Modulate antigenicity and fitness	(25)
HA	N27, N39, N181	Facilitate DC-SIGN binding	Enhance infection via the lectin receptor	(80)
Host glycoproteome	Global glycan remodeling	Reduces sialylation and alters trafficking	Facilitates viral release	(81)
HA	N54, N125, N160	Drive antigenic variation through glycosylation changes	Generate antigenic variants	(82)
HA	Added NLG sites	Increase HA incorporation into virions	Improve vaccine efficacy	(83)
HA	Variant-specific sites	Sequence variation alters glycosylation patterns	Influences antigenic evolution	(84)

### 6.2 Glycan shielding and immune escape

N-linked glycosylation of influenza virus surface glycoproteins functions as an important immune-evasion mechanism that modulates antigen exposure, receptor engagement, and host inflammatory responses. Changes in glycan occupancy, position, and density collectively influence the degree of “glycan shielding”,” allowing influenza viruses to evade both innate and adaptive immunity while maintaining infectivity. Perturbations in host glycosylation pathways may directly influence the viral glycome without requiring viral genomic changes. Viruses generated under low glycan occupancy exhibit reduced antibody production and susceptibility to collectin-mediated neutralization, suggesting that modulating HA and NA glycosylation density alone might improve immune escape potential (26). The results presented suggest that host metabolic or cellular conditions that might alter glycosylation efficiency may impact viral pathogenicity and vaccination responses.

**Figure 3:**
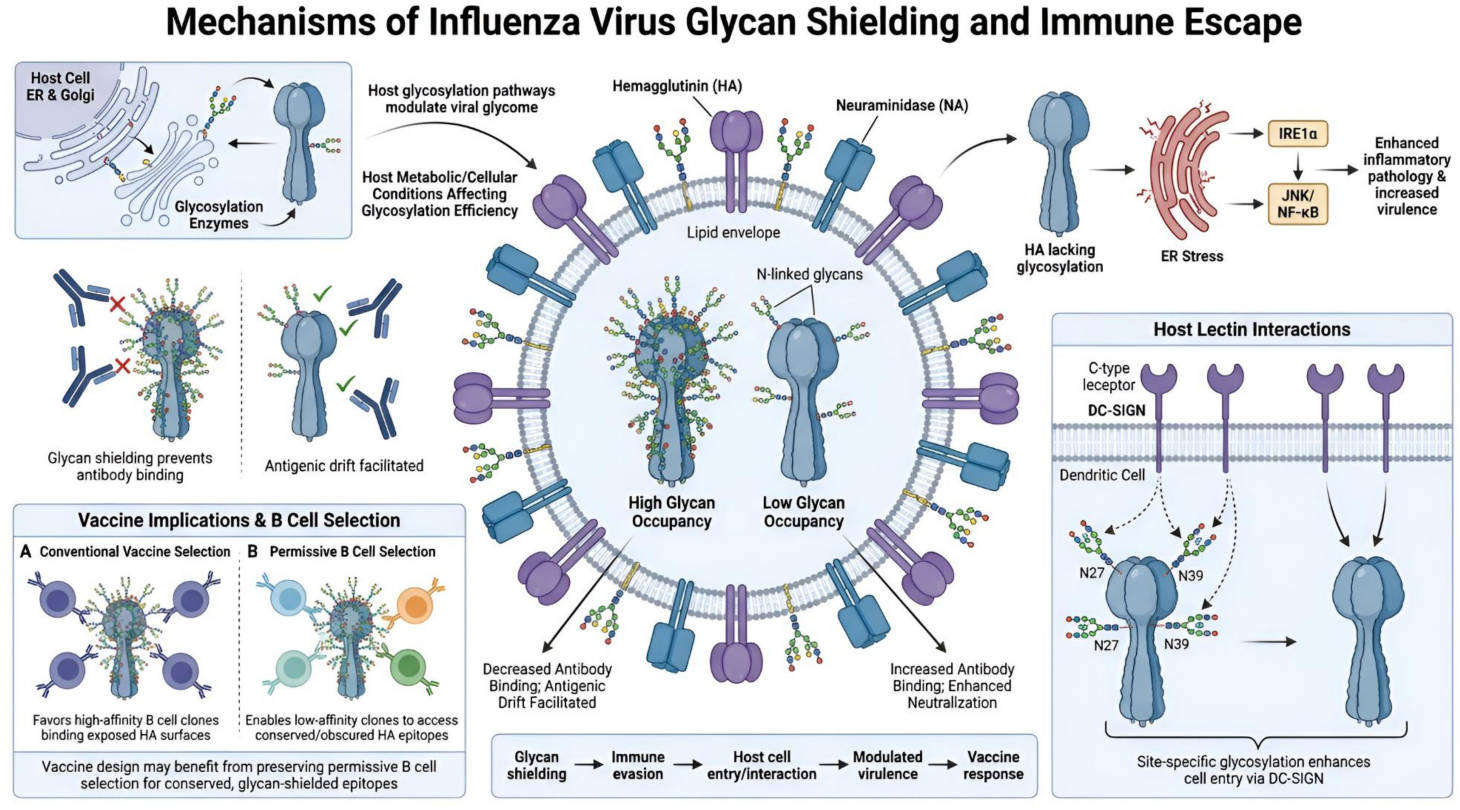
Mechanisms of influenza virus glycan shielding and immune escape. N-linked glycosylation of HA and NA promotes immune evasion by steric shielding of epitopes, facilitating antigenic drift, and enhancing entry via DC-SIGN (N27/N39). Host metabolic and glycosylation conditions alter viral glycan density without requiring genomic changes. Loss of HA head glycans triggers ER stress and IRE1α–JNK/NF-κB signaling, increasing inflammatory pathology and virulence. These processes have key implications for vaccine design through permissive B cell selection targeting glycan-shielded conserved epitopes.

In addition, these site-specific changes in HA glycan structures also affect immune recognition and virulence. The elimination of glycosylation sites in the HA head domain has been associated with increased endoplasmic reticulum stress, thereby activating inflammatory pathways, such as IRE1α-dependent JNK/NF-κB pathways, resulting in increased inflammatory pathology and increased virulence in mammalian hosts (79). On the other hand, the acquisition of additional glycan structures in the HA of influenza viruses sterically shields epitopes, thereby making it difficult for neutralizing antibodies to bind, a phenomenon that facilitates antigenic drift, depending on the avidity of the influenza viruses for their target host cells (25). Besides steric shielding, the glycan structures on influenza viruses interact with host lectin receptors, thereby affecting their entry into target cells. Glycosylation at N27 and N39 facilitates interaction with the dendritic cell lectin DC-SIGN, thereby enhancing entry into host cells. Mutation of these sites reduces entry via the lectin pathway (80). Glycan shielding is widely considered an important evolutionary adaptation strategy in influenza viruses, which dynamically adjust their glycosylation sites to balance immune evasion, entry, and virulence (**Figure 3**; **Table 2**). HA glycan shielding also has implications for vaccine-directed B cell selection. Although not studied in the influenza system, Ronsard et al. provided an important conceptual framework by showing that permissive B cell selection can allow low-affinity B cell receptor clones to persist and expand during immunization against a conserved HIV Env vaccine target (85). In a transgenic mouse model designed to mimic human antibody diversity and somatic hypermutation, sequential immunization with monomeric HIV Env immunogens focused B cell memory toward the conserved CD4-binding site through both conventional affinity maturation and reproducible expansion of low-affinity BCR clones. Notably, in some lineages, somatic hypermutation facilitated target acquisition by decreasing binding strength rather than increasing affinity. This finding suggests that vaccine strategies aimed at conserved but difficult-to-access viral epitopes may benefit from preserving permissive B cell selection, rather than exclusively favoring high-affinity responses to immunodominant exposed surfaces (85). By analogy, influenza vaccine design may need to consider how immunogen structure and boosting regimens can maintain or recruit B cell clonotypes capable of exploring conserved HA surfaces that are constrained by glycan shielding or conformational accessibility.

### 6.3 Glycosylation-driven strain evolution

Influenza virus evolution is influenced not only by amino-acid substitutions but also by dynamic remodeling of glycosylation patterns that alter antigenicity, receptor engagement, and host interactions. Increasing evidence indicates that glycosylation changes operate as a parallel evolutionary axis, rapidly generating phenotypic diversity without requiring major structural alterations in viral proteins. During infection, influenza viruses can substantially remodel the host glycoproteome, causing extensive desialylation and shortening of host glycans through neuraminidase activity and infection-induced disturbances in Golgi trafficking. These alterations alter the cellular glycan environment, influencing virion release, protein stability, and immunological signaling, thereby imposing selection pressures that affect strain development across multiple replication cycles (81). Variation in HA N-linked glycosylation sites is a crucial factor influencing antigenic drift at the viral level. Integrated analyses of sequence variation and glycosylation patterns show that mutations introducing or removing HA glycosylation sequons, particularly at residues positioned within or adjacent to antibody-binding epitopes, strongly correlate with the emergence of antigenic variants in circulating H1N1 viruses (82).

Experimental modification of glycosylation patterns indicates their evolutionary significance. The addition of defined N-glycosylation sites to the HA protein of H7N9 viruses increases HA incorporation into virions, improves viral growth in culture, and elicits broader cross-reactive antibody responses, indicating that glycosylation remodeling can influence viral fitness and immune recognition (83). High-resolution glycoproteomic analyses confirm that even minor sequence variations or differences in host expression systems result in measurable, site-specific changes in HA glycosylation profiles, demonstrating that glycan heterogeneity is an inherent and evolving feature of circulating viral strains (84). These results support a hypothesis in which glycosylation dynamics serve as a flexible evolutionary process that supports genetic mutation. By modulating antigenic surfaces, host glycan environments, and virion structural properties, glycosylation-driven diversification may enable influenza viruses to generate adaptive phenotypes over, facilitating immune escape, persistence, and the emergence of new epidemiologically successful strains (**Figure 3**, **Table 2**).

## 7 Acetylation, lipidation, methylation, ISGylation, and neddylation

### 7.1 Acetylation-dependent polymerase regulation

Lysine acetylation has emerged as an important post-translational modification that can influence the viral transcriptional machinery and host antiviral responses during influenza infection. Acetylation of polymerase-associated proteins has been reported to influence catalytic activity, RNA synthesis, and immune evasion. Regulation of the viral polymerase complex has been linked, in part, to acetylation of the PA subunit. Host acetyltransferases, such as PCAF and GCN5, acetylate the N-terminal domain of PA, and lysine 19 is identified as an important acetylation site. Acetylation has been reported to enhance PA endonuclease activity, a process important for efficient cap-snatching and RNA-dependent RNA polymerase activity. This modification is associated with increased PA endonuclease activity and improved cap-snatching efficiency and, consequently, increasing RNA-dependent RNA polymerase activity within the cell, illustrating the direct catalytic effect of acetylation on polymerase function (86). In direct complement to this, N-terminal acetylation of PA-X, a polymerase accessory protein, influences its nuclear localization and host shutoff activity, facilitating efficient host mRNA degradation and antiviral gene expression inhibition (87). These data show that, among PA-derived proteins, acetylation events play a significant role in facilitating transcriptional initiation and host repression.

Acetylation also influences viral replication indirectly by modulating critical immune antagonists that promote polymerase activity. Acetylation of NS1 at lysine 108 has been reported to enhance its ability to suppress type I interferon signaling pathways, including RIG-I-MAVS-TBK1-IRF3 signaling, thereby maintaining a cellular environment conducive to viral transcription and replication; loss of this acetylation reduces viral replication and virulence (23). Furthermore, the acetylation of structural glycoproteins, such as hemagglutinin, contributes to viral pathogenicity by enhancing HA stability and affecting replication efficiency. This highlights increased acetylation-dependent regulation of viral fitness, which indirectly supports polymerase-driven replication (18). Overall, our findings suggest a concept in which acetylation acts as a multilayered regulatory mechanism that coordinates influenza virus polymerase activity via direct enzymatic activation of polymerase components, regulation of host shutdown pathways, and enhanced immune antagonism. This acetylation-dependent regulation promotes effective viral transcription while inhibiting host antiviral responses, highlighting acetylation as a functionally important regulatory layer that can influence influenza efficiency. Importantly, the strength of evidence varies across acetylation sites and experimental models, and it remains to be established which acetylation events are consistently conserved and rate-limiting during infection *in vivo*.

### 7.2 Palmitoylation and membrane targeting

Protein S-palmitoylation is an important post-translational modification that contributes to membrane targeting, assembly, and infectivity of influenza viruses by controlling the lipid anchoring of viral and host proteins. The site-specific acylation of envelope proteins is important for their proper processing through the secretory pathway and for their stable incorporation into budding virus particles. HA is a key target of S-palmitoylation; acylation of the protein at specific cytoplasmic cysteine clusters is crucial for efficient viral replication. The absence of palmitoylation sites in influenza B virus HA has been reported to impair the formation of infectious particles, indicating that lipid modification is essential for membrane attachment and virion assembly (88). Multiple ZDHHC family palmitoyltransferases, including ZDHHC2, 8, 15, and 20, mediate HA and M2 acylation in influenza A virus, which co-localize with viral proteins along the exocytic pathway and have partially overlapping substrate specificities, thereby supporting robust acylation during infection (89). Evolutionary investigations reveal that conserved clusters of acylated cysteines persist among influenza viruses and their mammalian reservoirs, emphasizing the functional significance of S-acylation for viral fitness (90).

Beyond direct viral protein modification, influenza infection also modulates host acylation pathways to optimize replication. The viral NS1 protein induces upregulation of the host acyltransferase ZDHHC22, suggesting that viruses actively remodel host lipid-modification networks to support infection, even though this enzyme appears to target host substrates rather than HA or M2 directly (91). Palmitoylation can also influence viral pathogenicity *in vivo*; for example, acylation of the M2 protein is dispensable for replication in cultured cells but contributes to virulence in animal models, indicating that lipid modification has context-dependent functions (92). Host antiviral defenses are likewise regulated by palmitoylation. S-palmitoylation of interferon-induced transmembrane (IFITM) proteins controls their interaction with cholesterol in membranes and determines the specificity of antiviral restriction against influenza virus, illustrating how lipid modification governs both viral and host membrane-associated processes (93). Collectively, palmitoylation functions as a central regulator of membrane targeting and viral assembly by coordinating lipid anchoring of viral proteins, remodeling host acyltransferase pathways, and modulating membrane-dependent antiviral defenses. These multilayered roles position protein acylation as a conserved determinant of influenza virus replication efficiency and pathogenic potential.

### 7.3 RNA methylation and viral gene expression

RNA methylation represents an important layer of epitranscriptomic regulation, playing a critical role in controlling influenza virus gene expression by regulating RNA processing, vRNP assembly, and polymerase activity. The methylation pathways of both viral and host origin play a role in fine-tuning transcriptional efficiency and genome packaging during the infectious process. The N6-methyladenosine (m6A) modification of viral RNA has a direct positive effect on its function. m6A modification of viral genomic RNA has been reported to facilitate interactions between vRNA and polymerase proteins for vRNP assembly, and is associated with enhanced viral gene expression and pathogenic potential; however, the absence of m6A methylation motifs impairs polymerase activity and attenuates viral replication *in vitro* and *in vivo* (94). In addition to the structural role of m6A modification during the assembly of vRNP, m6A modification also regulates mRNA processing of the influenza virus. The NS1 protein of the influenza virus regulates autoregulation of NS segment splicing through m6A modification of a conserved site of the NS segment of the viral genome through the recruitment of the host cell protein YTHDC1, a nuclear reader of m6A modification; this autoregulation balances the levels of both spliced and unspliced viral mRNA for efficient replication (95).

Cytosine methylation serves as an additional regulatory mechanism. The host methyltransferase NSUN2 installs 5-methylcytosine (m5C) on both positive- and negative-strand viral RNAs, and NSUN2 depletion reduces correct genomic segment packing, leading to more defective interfering particles and reduced viral pathogenicity (96). Influenza infection also alters m5C profiles across host long noncoding RNAs, affecting the regulation of pathways involved in pathogen identification and cellular stress responses, indicating that epitranscriptomic remodeling of host RNAs contributes to virus-host interactions (97). Protein methylation is further integrated with RNA methylation mechanisms to control viral transcription. The host enzyme PRMT5 symmetrically dimethylates arginine residues on the PB2 subunit of the polymerase, increasing polymerase activity and facilitating viral RNA production (98). Overall, our results show that coordinated RNA and protein methylation processes control influenza gene expression at various levels, including RNA processing, vRNP assembly, genome packing, and polymerase activity. This multilevel epitranscriptomic control identifies RNA methylation as a key factor of influenza replication efficiency and pathogenicity.

### 7.4 ISGylation and antiviral restriction

ISGylation, the covalent modification of proteins by the ubiquitin-like protein ISG15, is a multifaceted antiviral response pathway that encompasses intracellular restriction, cytokine responses, and tissue-protective mechanisms during influenza virus infection. In contrast to ubiquitin modification, ISGylation is generally thought to have more indirect effects on viral replication kinetics. ISGylation plays a protective role during influenza virus infection by modulating the host response through the conjugation of ISG15 to other proteins, suggesting an immunomodulatory role for ISGylation during influenza infection (99). ISGylation can be mediated by various antiviral cofactors during restriction at the cell level. ISG15 has been reported to restrict viral replication, in part through interaction with the RNA helicase DDX6, which binds the viral nucleoprotein, inhibits viral polymerase activity and nuclear import of the nucleoprotein, and enhances the type I interferon response and the levels of free ISG15 conjugates (37).

In addition to its intracellular role in protein modification, ISG15 also exerts an extracellular immunomodulatory effect as a cytokine-like protein. In its extracellular role, ISG15 acts via the LFA-1 integrin to induce IFN-γ secretion in lymphocytes and NK cells, thereby connecting early innate immunity with later adaptive immunity; in its extracellular role, the effector proteins of the virus can interfere with the process of ISG15 secretion and its de-conjugation, which represents the interface between the virus’s immune evasion mechanisms and ISGylation signaling (100). In certain disease models, ISG15 signaling modulates disease-related inflammatory pathways; in the case of influenza infection, activation of the TNF-α-ISG15-EGR1 axis modulates ferroptosis-related transcriptional pathways (101). These data suggest that ISGylation represents a multifaceted antiviral response to influenza virus infection that limits infection in the host through intracellular protein modification and extracellular signaling and inflammatory pathways.

### 7.5 Neddylation-mediated regulation of viral proteins

Neddylation, defined as the covalent modification of the ubiquitin analog protein NEDD8 with target substrates, has now been identified as an additional mechanism regulating influenza virus replication by controlling the levels and activities of major viral proteins. During influenza infections, neddylation of host cells is initiated, and pharmacologic inhibition of this process has been shown to significantly reduce replication of diverse influenza virus subtypes by interrupting the Cullin-RING ligase-dependent and NF-κB-related inflammatory pathways (102). In regulating influenza viral proteins, the polymerase subunit PB2 undergoes direct neddylation at K699 by the E3 ligase HDM2, thereby limiting viral replication by decreasing polymerase stability. In contrast, neddylation-deficient mutants of the PB2 protein exhibit increased protein stability, enhanced reproductive efficiency, and increased virulence *in vivo* (103). Neddylation of another major influenza protein, M1, at K187 by HDM2 limits influenza reproduction by extending the half-life of the M1 protein. It acts as an additional host restriction mechanism on the reproduction of the influenza virus (104). Collectively, these studies suggest that neddylation may function as a negative regulator of influenza virus infection by destabilizing essential viral proteins and modulating host signaling pathways. Targeting the neddylation machinery, therefore, may represent a potential antiviral strategy that can simultaneously impair viral replication and limit infection-associated inflammatory responses.

## 8 Host PTM signaling networks exploited by Influenza virus

IAV engages complex PTM networks to reprogram host signaling pathways in ways that can support viral replication, immune evasion, and pathogenesis. Through coordinated modulation of phosphorylation, ubiquitination, and proteolysis, IAV can couple viral fitness with host inflammatory and cell-death pathways. This review evaluates key host variables and PTM nodes engaged by IAV, categorizing them as pro-viral mechanisms (**Table 3**) that enhance replication and anti-viral mechanisms (**Table 4**) that inhibit infection. MAPK modules (ERK/JNK/p38), TAK1 as a central survival-inflammation switch, JAK-STAT signaling, cytoskeleton/contractility regulators (MLC, JIP4, cortactin), metabolic rewiring (SREBP2/HMGCR, CRBN/AMPK), ubiquitin ligases (TRIM25, TRIM31, USP1, ASB3), and caspase-mediated programs (apoptosis, necroptosis, pyroptosis) will be discussed. The next sections discuss inflammasome priming and transcriptional regulation via NF-κB, AP-1/cFos, and the STAT axis. Collectively, available evidence suggests that PTM-targeted approaches may help distinguish viral exploitation from immunopathology (**Table 5**).

**Table 3:** Host signaling pathways modulate influenza replication, immunity, and disease outcomes.

Host factor/mechanism	Pro-viral role	Outcome/key finding	Ref
MKP5 dephosphorylation of IRF3	Suppresses IRF3 activation	Reduced Type I IFN response, enhanced viral evasion	(32)
TAK1-JNK activation leading to autophagy	Facilitates virus-induced autophagy	Increased virus replication in H5N1	(105)
CXCL8-MAPK-hnRNP-K translocation	Enhances recognition of viral RNA	Facilitated replication of EV-D68, rhinovirus, IAV	(106)
Atypical p38-TAB1 interaction	Drives pulmonary injury	Increased lung damage and monocyte recruitment	(107)
ERK-dependent PAI-1 upregulation	Disrupts PAI-1/tPA balance	Impaired neuronal development via paracrine effects	(108)
PKCα-MEK1-ERK2 complex-NP phosphorylation	Promotes nuclear export of vRNPs	Enhanced virus propagation	(28)
TAK1-IKK/p38/MK2 inhibition of RIPK1	Suppresses apoptosis and necroptosis	Prolonged cell survival for viral replication	(41)
TRIF-TAK1 suppression of RIPK1/caspase-8/3	Inhibits pyroptosis	Enhanced virus replication in epithelial cells	(42)
p-STAT1 increased vRNA	Supports viral replication	Elevated inflammatory cytokines and viral burden	(109)
GBP7 suppression of NF-κB/JAK-STAT	Suppresses IFN and cytokines	Facilitated IAV replication	(110)
High-dose H1N1 dysregulated STAT1/3	Induces imbalanced immune responses	Pulmonary immunopathological damage	(111)
STAT3-SREBP2/HMGCR cholesterol biosynthesis	Increases cellular cholesterol	Enhanced virus endocytosis and replication	(30)
JAK-STAT activators (IFN/IL-6) higher in juveniles	Drives inflammation	Severe pathologies in juvenile mice	(112)
JAK inhibitors suppress IFN	Removes innate barriers	Facilitates viral propagation	(113)
TAK1-RORγ HMGCR upregulation	Reprograms cholesterol biosynthesis	Facilitated virus replication	(15)
CRBN AMPK ubiquitination/degradation	Shifts metabolism to anabolism	Favors viral replication via lipid droplets	(61)
High humidity/temp gut flora/Nod/RIP2/NF-κB	Aggravates immune imbalance	Worsened influenza infection	(114)
PB1-F2 mtDNA release/cGAS-STING-NF-κB	Mediates inflammation	Severe disease in elderly	(115)
NS1 hijacks NF-κB for type III IFN	Represses antiviral response	Reduced IFN expression	(116)
PA-X NF-κB inhibition	Counteracts innate responses	Suppressed immunity	(117)
cFos reduces apoptosis/IFN-β	Regulates replication via nuclear function	Enhanced viral proliferation	(118)
AP-1 pro-IL-1β transcription/NLRP3	Facilitates inflammasome activation	Increased inflammation	(119)
NF-kB boosts lung TRM maintenance	Enhances TRM for pathogen control	Dual role in memory, but pro-viral in infection context	(120)
IL-17RA-NF-κB disrupts tolerance	Promotes bacterial invasion	Increased dissemination during co-infection	(121)

**Table 4:** Host antiviral compounds and signaling regulators modulate influenza replication, inflammation, and immune responses.

Host factor/compound	Anti-viral role	Outcome/key finding	Ref
Theaflavin-3’-gallate (TF2b)	Downregulates TLR4/MAPK/p38	Inhibits replication, reduces cytokines and lung injury	(122)
Miquelianin	Inhibits MAPK signaling	Inhibits replication, reduces IL-6/IL-1β and lung injury	(123)
Shu-Feng-Jie-Biao Formula (SFJBF)	Inhibits NF-κB/ERK MAPK	Ameliorates lung injury, suppresses overactivated immunity	(124)
Caspase-8 cleavage of CYLD	Enhances RIG-I/TAK1 ubiquitination	Boosts innate antiviral immunity	(125)
β-sitosterol	Disrupts RIG-I/IFN/STAT signaling	Reduces proinflammatory response, protects from lung injury	(126)
Early p-STAT2 (RIG-I/MAVS dependent)	Boosts ISGs expression	Critical for early innate antiviral immunity	(31)
Haoqin Qingdan Tang (HQQDT)	Regulates JAK/STAT pathway	Inhibits replication, reduces cytokines	(127)
Yinchenhao Tang (YCHT)	Inhibits JAK/STAT signaling	Inhibits replication, reduces inflammation	(128)
Inhibition of MLC phosphorylation (RhoA/PKC/ERK)	Restricts vRNP nuclear export	Limits viral replication	(129)
p-JIP4 (S730)	Reduces viral polymerase activity	Inhibits replication	(130)
NFkB pathway	Maintains lung TRM	Enhances antiviral memory	(120)
HSL-2	Interacts with PPAR-γ/NF-κB	Inhibits infection, attenuates inflammation	(131)
Artemisia Extracts	Targets HA/NA, modulates TLR4/MyD88/NF-κB	Suppresses H1N1 infection	(132)
Artemisia annua L. leaf extracts (AALME)	Targets NP, blocks mitochondrial apoptosis	Suppresses replication, reduces pathogenicity	(133)
Andrographolide	Inhibits NF-κB/JAK-STAT	Reduces inflammation, inhibits replication	(134)
Baicalin	Inhibits caspase-3/GSDME	Suppresses pyroptosis, enhances survival	(135)
14-Deoxy-11,12-didehydroandrographolide (DAP)	Inhibits caspase-9 intrinsic pathway	Inhibits apoptosis, contributes to antiviral activity	(136)
H6N6-induced NLRP3/caspase-1/GSDMD	Induces pyroptosis in M1 macrophages	Host defense mechanism	(137)
GSDME deficiency	Reduces cytokines/inflammation	Limits lung damage, improves survival	(138)
Caspase cleavage of M2	Disrupts M2-LC3 interaction	Regulates virion production	(139)
Diethylcarbamazine (DEC)	Regulates IFN-β/IL-8	Reduces viral titer, modulates cytokines	(140)
miR-4776 targeting NFκBIB	Modulates NF-κB activation	Potential antiviral modulation	(141)

**Table 5:** Post-translational and RNA modifications modulate influenza virus replication and host antiviral responses.

PTM type	Target (protein/RNA)	Modified residue/site	Mechanistic effect	Impact on viral infection	Ref.
Acetylation	NS1	K108	Enhances IFN antagonism	Promotes replication and virulence	(23)
Acetylation	PA	K19	Enhances endonuclease and polymerase activity	Promotes replication	(86)
Acetylation	HA	K157/K169/K418/K459	Regulates HA stability and pathogenicity	Controls virulence and vaccine attenuation	(18)
N-terminal acetylation	PA-X	N-terminus	Controls nuclear localization and host shutoff	Enhances immune evasion	(142)
Palmitoylation	HA (Influenza B)	Cysteine residues	Required for virion formation	Essential for replication	(88)
Palmitoylation	M2	C50	Modulates virulence without affecting replication in vitro	Contributes to in vivo pathogenicity	(92)
S-acylation regulation	Host ZDHHC22 pathway	Host acyltransferase	NS1-induced upregulation of acylation enzymes	Modulates host lipid modification pathways	(91)
Palmitoylation	HA/M2	Multiple cysteines	ZDHHC2/8/15/20 mediates acylation	Required for HA/M2 lipid modification	(89)
Palmitoylation	IFITM proteins	Cysteine residues	Regulates cholesterol interaction	Controls antiviral specificity	(93)
S-acylation	HA/M2	Conserved cysteine clusters	Essential lipid modification across influenza viruses	Supports replication	(90)
m6A RNA methylation	NS segment mRNA	A385	Regulates splicing via YTHDC1 recruitment	Enhances replication efficiency	(95)
m5C RNA methylation	vRNA	Multiple sites	Regulates genome packaging and particle formation	Required for infectious virions	(96)
m5C RNA methylation	Host lncRNAs	Transcriptome-wide sites	Modulates host antiviral responses	Regulates host–virus interaction	(97)
Arginine methylation	PB2	RG motifs	Enhances polymerase activity	Promotes viral replication	(98)
m6A RNA methylation	vRNA	Multiple motifs	Enhances vRNA–polymerase interaction	Promotes replication and pathogenicity	(94)
ISGylation	Host/viral proteins	ISG15 conjugation	Protects against virus-induced lethality	Modulates disease severity	(99)
ISG15 regulation	NP–DDX6 pathway	ISG15-dependent signaling	Enhances interferon responses	Restricts replication	(37)
ISG15 signaling	Extracellular ISG15	Secreted ISG15	Cytokine-like immune signaling	Modulates immune responses	(100)
ISG15 pathway	TNF-α/ISG15/EGR1 axis	Signaling pathway	Induces ferroptosis in trophoblasts	Contributes to pathology	(101)
Neddylation	Host CRL/NF-κB pathway	NEDD8 pathway activation	Promotes inflammatory signaling	Enhances replication	(102)
Neddylation	PB2	K699	Reduces protein stability	Restricts viral replication	(103)
Neddylation	M1	K187	Reduces the stability of M1	Restricts replication	(104)

### 8.1 Kinase-driven phosphorylation signaling pathways in influenza infection

#### 8.1.1 MAPK signaling rewiring and inflammation/replication coupling

IAV infection has been shown to alter host mitogen-activated protein kinase (MAPK) signaling pathways, including the ERK, JNK, and p38 modules, linking viral replication to inflammatory responses. These rewiring events involve PTMs such as phosphorylation and are thought to help influenza viruses evade antiviral immunity while supporting progeny virus production. For example, IAV increases the production of MAPK phosphatase 5 (MKP5), a dual-specificity phosphatase that dephosphorylates IRF3, reducing type I interferon (IFN) responses and promoting H1N1 and other RNA virus replication (32). The NS1 protein of H5N1 IAV stimulates TGF-β-activated kinase 1 (TAK1), leading to JNK phosphorylation and increased autophagy, which favors viral replication. Inhibiting TAK1 prevents this cascade, highlighting its involvement in the replication-inflammation nexus (105). Theaflavin-3’-gallate (TF2b), a member of black tea’s active compound family, inhibits H1N1 replication by downregulating TLR4, MAPK, and p38, thereby reducing pro-inflammatory cytokines (IL-6, TNF-α, IL-1β) and chemokines (CXCL2, CCL3), relieving pneumonia without direct antiviral activity (122). Miquelianin, a flavonoid, downregulates H1N1-induced MAPK, reducing severe inflammation (IL-6, TNF-α, IL-1β) and chemokines (CXCL2, CCL3), and relieving pneumonia without direct antiviral effects (122). Miquelianin, a flavonoid, inhibits H1N1-induced MAPK activation, reducing cytokine storms (IL-6, IL-1β) and lung damage *in vivo*. This demonstrates how PTM-targeted therapies may separate replication from hyperinflammation (123).

IAV also utilizes the chemokine-associated MAPK signaling pathway to modulate host susceptibility. CXCL8 acts on CXCR1/2 receptors to activate the MAPK pathway, leading to the translocation of hnRNP-K to the cytoplasm, where it binds to viral RNA to enhance 5’UTR activity and replication; this pathway also applies to rhinovirus and enterovirus infections and represents a common pathway for susceptibility to these viruses (106). Atypical activation of the p38 pathway through TAB1 interaction contributes to acute lung injury (ALI) progression during IAV infection, irrespective of viral loads; disruption of the TAB1–p38 pathway accelerates monocyte recruitment and alleviates ALI (107). Traditional remedies such as the Shu-Feng-Jie-Biao formula (SFJBF) inhibit ERK1/2 and NF-κB p65 phosphorylation, reducing neutrophil infiltration and the subsequent release of inflammatory cytokines (MIP-2, TNF-α, MCP-1), thereby conferring protection from H1N1-induced ALI (124). Finally, in CNS contexts, IAV activates astrocytic ERK to induce plasminogen activator inhibitor-1 (PAI-1), suppressing tPA activity and impairing neuronal outgrowth via paracrine effects (108). Collectively, these investigations demonstrate how IAV-induced MAPK PTMs reorganize signaling to combine replication machinery and inflammatory amplification, providing targets for broad-spectrum antivirals.

#### 8.1.2 TAK1 as a central switch integrating survival, death, and inflammation

TAK1 has emerged as an important regulatory node in IAV pathogenes, coordinating PTMs to balance cell survival, programmed cell death, and inflammatory responses. IAV infection has been shown to induce TAK1 phosphorylation in alveolar epithelial and fibroblast cells, activating downstream IKK and p38/MK2 pathways. These effectors phosphorylate RIPK1 at inhibitory sites (Ser25 via IKK and Ser321 via p38/MK2), suppressing RIPK1-dependent apoptosis and necroptosis. Consequently, TAK1 inhibition has been associated with enhanced caspase-8/3 activation and increased cell death, alongside reduced viral titers in multiple experimental models, thereby prolonging mouse survival during H1N1 infection (41). Paradoxically, IAV stimulates caspase-8 not only to induce cell death but also to boost antiviral immunity. Caspase-8 cleaves the deubiquitinase CYLD, preventing the removal of K63-linked ubiquitin chains from TAK1 and RIG-I. This promotes TAK1 ubiquitination, TBK1-IRF3 phosphorylation, NF-κB activation, type I interferon (IFN-β) production, and antiviral gene expression (MX1, ISG15). Caspase-8 inhibitors (Z-VAD, Z-IETD) or knockouts reduce these responses, enhancing viral replication across the H5N1, H5N6, and H1N1 subtypes (125). Furthermore, TRIF-TAK1 signaling prevents caspase-8/3-induced pyroptosis by inhibiting GSDMD/E cleavage in airway epithelia. ZBP1 and RIPK1, but not RIPK3, are required for this activation; TAK1 inhibition increases pyroptosis, while GSDMD/E, ZBP1, or RIPK1 knockouts prevent it but boost replication. In mice, TAK1 inhibition increases lung pyroptotic markers (42). TAK1 links pro-survival PTMs with inflammatory restriction, avoiding excessive death and promoting IAV proliferation, underlining its therapeutic potential for dissociating viral exploitation from host disease.

#### 8.1.3 JAK–STAT signaling as phosphorylation-driven control of antiviral state and pathology

Janus kinase-signal transducer and activator of transcription (JAK-STAT) signaling acts as a phosphorylation-dependent mechanism that dynamically controls antiviral states, inflammatory amplification, and pathological outcomes through PTMs of STAT proteins. Upstream of STAT phosphorylation, cytokine availability itself represents an important regulatory checkpoint for JAK–STAT signaling. Yousif et al. showed that conventional dendritic cells are a major source of circulating soluble IL-6 receptor (sIL-6R) and thereby systemically regulate IL-6 signaling by setting the in-solution persistence of IL-6 (143). This cDC-derived sIL-6R axis forms part of a blood cytokine-buffering system that shapes IL-6 bioavailability before intracellular JAK–STAT activation occurs (143). Therefore, influenza-associated JAK–STAT responses should be interpreted not only through downstream STAT phosphorylation but also through upstream cytokine-buffering mechanisms that determine the magnitude and duration of cytokine signaling.

IAV-induced STAT1 phosphorylation has been associated with both proviral and pro-inflammatory effects, and phosphoproteomics analysis of H5N1 infection in A549 cells revealed that p-STAT1 levels were upregulated, and its inhibition by fludarabine reduced progeny titers, vRNA, and cytokines (IL-6, TNF-α), but increased survival and reduced lung pathology in H5N1-infected mice, indicating its pro-viral effect (109). Guanylate binding protein 7 (GBP7), upregulated in IAV infection in lung, PBMCs, and A549 cells, has pro-viral and pro-inflammatory effects by inhibiting type I/III IFNs and cytokines through JAK/STAT and NF-κB pathways, and its knockout increases levels of p-STAT1/2, IFN-β, and antiviral ISGs, restricting replication, whereas overexpression facilitates it (110).

β-Sitosterol relieves IAV-induced inflammation by inhibiting RIG-I/JAK/STAT signal cross-talk and decreasing p-STAT1, NF-κB, and p38 MAPK activation, thereby terminating the amplification of IFN and the apoptosis of alveolar epithelial cells; in mice, β-Sitosterol relieves lung injury, cytotoxic T cell recruitment, and lethality (126). IAV infection, in a dose-dependent manner, disrupts innate immunity by inducing sustained activation of p-STAT1/3; high-dose pH1N1 IAV infection causes excessive cytokines, neutrophil recruitment, and M1 macrophage polarization, resulting in acute lung injury (ALI), and inhibiting STAT1/3 relieves the pathology in low-dose IAV infection models (111). IAV has been reported to activate STAT3, which in turn can upregulate SREBP2via JAK2, increasing HMGCR expression and cholesterol production for virion assembly; STAT3/JAK2 knockout or inhibition (S3I-201) lowers cholesterol, binding, and replication, which is reversible with exogenous supplementation (30).

**Figure 4:**
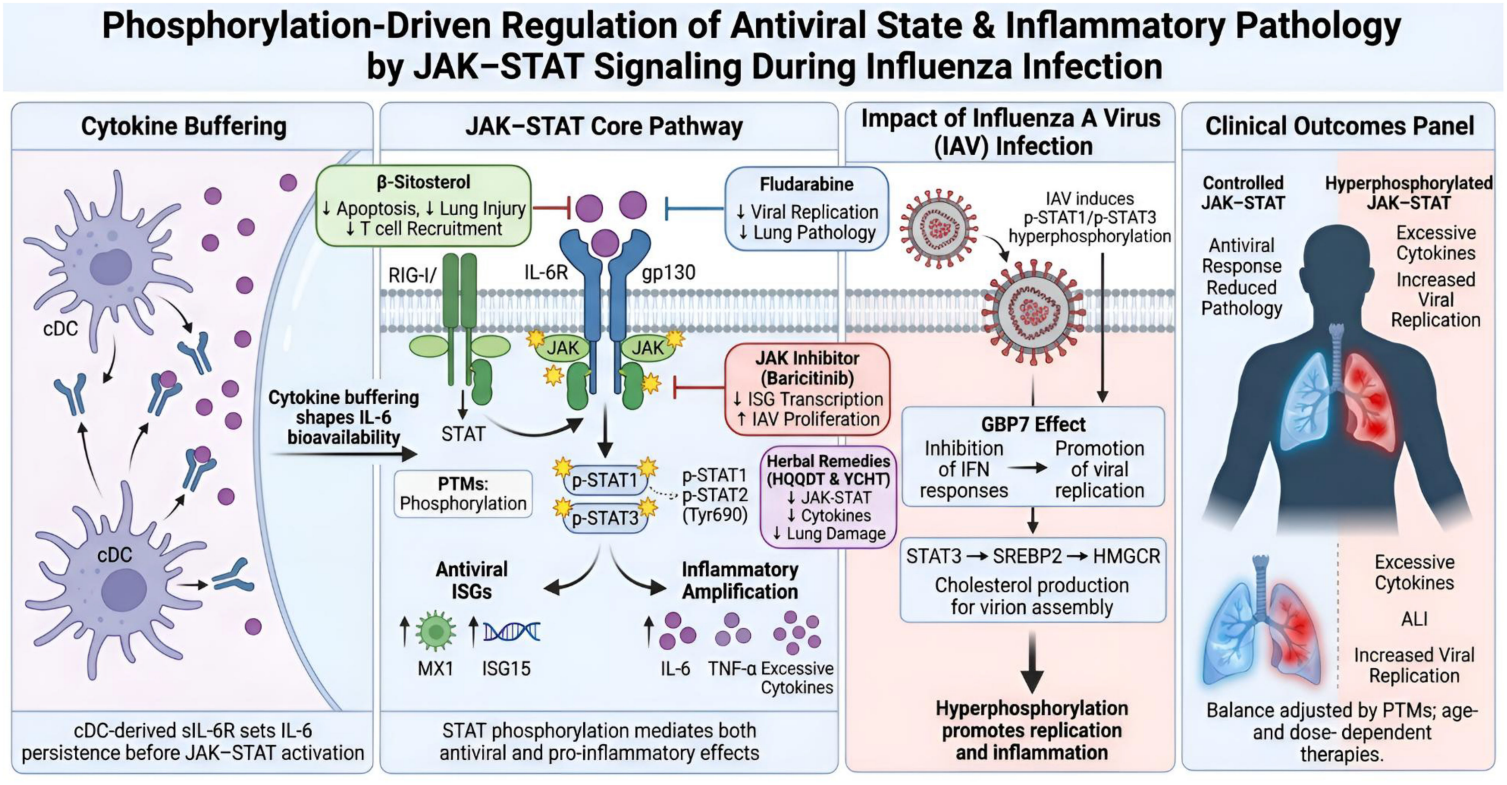
Phosphorylation-driven regulation of antiviral state and inflammatory pathology by JAK–STAT signaling during influenza A virus (IAV) infection. Upstream cytokine buffering by cDC-derived sIL-6R shapes IL-6 bioavailability before JAK–STAT activation, while downstream STAT phosphorylation (p-STAT1/2/3) mediates both protective ISG induction and pro-inflammatory amplification including SREBP2-HMGCR-mediated cholesterol synthesis for virion assembly. Hyperphosphorylation skews this axis toward cytokine storm, acute lung injury, and enhanced viral replication, highlighting opportunities for dose- and age-adapted therapeutic strategies with JAK inhibitors and herbal remedies.

Initial STAT2 Tyr690 phosphorylation, independent of type I IFNs, stimulates early antiviral immunity via RIG-I/MAVS; MAPK12 and Syk orchestrate this, and blocking them reduces ISGs (MX1, ISG15), enhancing replication (31). In mice, Haoqin Qingdan Tang (HQQDT) reduces H1N1/H3N2 replication by downregulating JAK-STAT, p-STAT1/3, IRF3, and cytokines (IL-6, IL-1β, IFN-β), thereby reducing lung damage (127). Mathematical modeling of juvenile IAV infection implicates enhanced JAK-STAT activators (type I IFNs, IL-6) in severe pathology, implying that age-specific IFN/IL-6 production promotes inflammation without inhibiting replication (112). Yinchenhao Tang (YCHT) regulates JAK-STAT signaling, reducing cytokine levels and pneumonia in H1N1 models, and 26 serum-active chemicals were identified using UPLC-Q-Orbitrap HRMS (128). JAK inhibitors, such as baricitinib, reduce ISG transcription, which promotes IAV proliferation by weakening IFN responses across strains (113). Overall, JAK-STAT PTMs control an adjustable antiviral-pathology balance in which hyperphosphorylation promotes replication and inflammation, allowing for age- and dose-specific therapies (**Figure 4**).

#### 8.1.4 Cytoskeleton and contractility phosphorylation nodes with antiviral potential

IAV infection can regulate cytoskeletal dynamics through phosphorylation-dependent post-translational modifications. These are potential targets for combating viral infections. IAV activates RhoA/Rho kinase, phospholipase C/protein kinase C, and HRas/Raf/MEK/ERK signaling cascades to regulate myosin light chain phosphorylation to modify the actin cytoskeleton. Inhibition of this phosphorylation by disrupting these signaling cascades, either genetically or chemically, blocks viral nuclear export of ribonucleoproteins and viral production, and, conversely, hyperphosphorylation of myosin light chain and activation of ERK enhance viral production, suggesting that it plays a pro-viral role (129).

JNK-interacting protein 4 (JIP4), a scaffold for MAPK signaling, is dynamically phosphorylated at Ser730 during IAV infection. JIP4 deletion increases viral titers and polymerase activity during the first cycle, whereas overexpression reduces both. This antiviral activity is independent of JNK/p38 regulation or interferon induction, but it directly inhibits viral RNA synthesis. S730 phosphorylation is required, since non-phosphorylatable mutants lose inhibitory efficacy, establishing JIP4 as a phosphorylation-gated antiviral restrictor (130). Phosphoproteomic studies during IAV entry identified virus-induced filopodia formation mediated by Cdc42 signaling to facilitate endocytosis. The host response to IAV entry inhibition involves the phosphorylation of cortactin at the cortex to limit actin polymerization and hence filopodia formation. Silencing or modifying the expression of factors that influence its phosphorylation enhances IAV entry efficiency into host cells (54). These PTMs represent important regulatory nodes where the balance of phosphorylation events may influence pro- and anti-IAV outcomes, suggesting their potential for disrupting IAV lifecycle events.

### 8.2 PTM-driven metabolic rewiring

The IAV infection manipulates PTMs to repurpose lipid metabolism, especially the cholesterol biosynthesis pathway, to support viral envelope formation, entry, and replication. This process is thought to involve integration of signaling cascades that increase sterol production, indicating a weakness in host lipid metabolism that can be exploited as a treatment target. IAV activates the JAK2-dependent phosphorylation of the transcription factor STAT3 on Tyr705, which promotes the nuclear translocation and transcriptional activation of SREBP2. SREBP2, in turn, regulates the expression of 3-hydroxy-3-methylglutaryl-CoA reductase (HMGCR), the rate-limiting enzyme in the cholesterol pathway that catalyzes mevalonate production. Genetic knockout or pharmacological inhibition of STAT3 (S3I-201) or JAK2 suppresses SREBP2/HMGCR induction, lowers cellular cholesterol content, impairs viral binding and endocytosis, and reduces replication across subtypes; however, exogenous cholesterol supplementation corrects these defects, confirming cholesterol’s essential pro-viral function in membrane dynamics and virion budding (30).

Simultaneously, IAV activates TAK1, which phosphorylates JNK and IKK, thereby activating AP-1 and NF-κB, which in turn induce RORγ transcription. RORγ co-activates SREBP2 to induce HMGCR transcription. RORγ deficiency or inverse agonists XY018 and GSK805 abolish HMGCR up-regulation, cholesterol synthesis, and viral titers *in vitro*. In contrast, RORγ knockout mice exhibit less viral replication, inflammation, weight loss, and mortality *in vivo*. XY018 protects these mice through mechanisms similar to those in RORγ deficiency. These results indicate that TAK1-RORγ PTMs are a pro-viral axis (15). Furthermore, cereblon (CRBN), an adaptor protein for the CRL4 E3 ligase complex, promotes IAV and influenza B virus replication by K48-linked ubiquitination and subsequent proteasomal degradation of AMP-activated protein kinase (AMPK). AMPK deficiency causes a shift from catabolic to anabolic metabolism, leading to lipid droplet accumulation for viral assembly. CRBN deficiency abolishes AMPK degradation and lipid droplets and restricts viral replication *in vivo*. Immunomodulatory drugs containing the imide ring structure (thalidomide analogs) inhibit CRBN, phenocopying CRBN deficiency, and restrict viral replication *in vivo* (61). These PTMs appear to converge on cholesterol and lipid metabolic pathways that support IAV propagation while avoiding metabolic checks. Targeting these pathways may provide new avenues to combat viral propagation by exploiting the host cell to circumvent the development of resistance to antiviral drugs.

### 8.3 Ubiquitin-mediated regulation of innate immune signaling

#### 8.3.1 RIG-I–MAVS ubiquitination

Ubiquitin-dependent regulation of the RIG-I/MAVS axis is widely recognized as an important contributor to antiviral interferon production during influenza virus infection, with host and viral factors dynamically regulating ubiquitination events to modulate this pathway. Different ubiquitin linkages involving RIG-I, MAVS, and adaptor proteins have been reported to play important roles in modulating innate antiviral signaling. Various host E3 ligases have been shown to enhance antiviral interferon production through ubiquitination-dependent mechanisms. TRIM22 mediates K63-linked ubiquitination of MAVS, a modification that has been associated with enhanced recruitment of TBK1 and IRF3 and increased interferon production while preventing MAVS-NLRX1 complex formation, which is inhibitory to MAVS (144). TRIM31 also regulates MAVS-dependent interferon production. However, influenza virus proteins can competitively bind TRIM31 to inhibit MAVS activation while simultaneously exploiting TRIM31-mediated ubiquitination of viral proteins to modulate replication (145). Upstream regulation also plays important roles in antiviral interferon production, with USP1-mediated deubiquitination of RIG-I has been reported to modulate protein stability and enhance interferon production, thereby contributing to restriction of influenza virus replication (146). Other pathways also enhance antiviral signaling by activating adaptor molecules. Induction of circRNA hsa_circ_0008085 has been reported to regulate TRAF6 expression by sequestering miR-146a-5p, thereby indirectly enhancing antiviral interferon production to inhibit influenza virus replication (147).

However, influenza viruses have evolved multiple strategies to counteract these antiviral ubiquitin-dependent processes. For example, Avian influenza viruses have been reported to use NS1 to inhibit TRIM25-mediated ubiquitination of human RIG-I but do little to inhibit TRIM25-mediated ubiquitination of duck RIG-I, suggesting that different species may use different mechanisms to evade the host immune response (33). Influenza polymerase subunits may also inhibit MAVS activity, in part by recruiting TRIM25, which facilitates K48-linked ubiquitination and autophagic degradation of MAVS, thereby affecting the activity of TBK1 and IRF3 (148). The host’s negative regulation of innate immunity also involves several proteins that modulate MAVS activity. For example, the E3 ligase ASB3 has been reported to facilitate K48-linked ubiquitination of MAVS at lysine 297, leading to its proteolytic degradation and, in turn, affecting interferon production (149). In addition, the ubiquitination of MAVS by TRIM25 during RIG-I-mediated activation has been implicated as a mechanism regulating the proteolytic turnover of MAVS complexes to facilitate the subsequent release of signaling complexes and thus properly activate IRF3 (34). Furthermore, the ubiquitination of TRAF6 by TRIM37 has also been implicated in the negative regulation of inflammation (150). TRIM25 may also prevent influenza replication via ubiquitination-independent RNA-binding processes that degrade viral mRNAs, demonstrating the multifunctionality of innate immune regulators (151).

**Figure 5:**
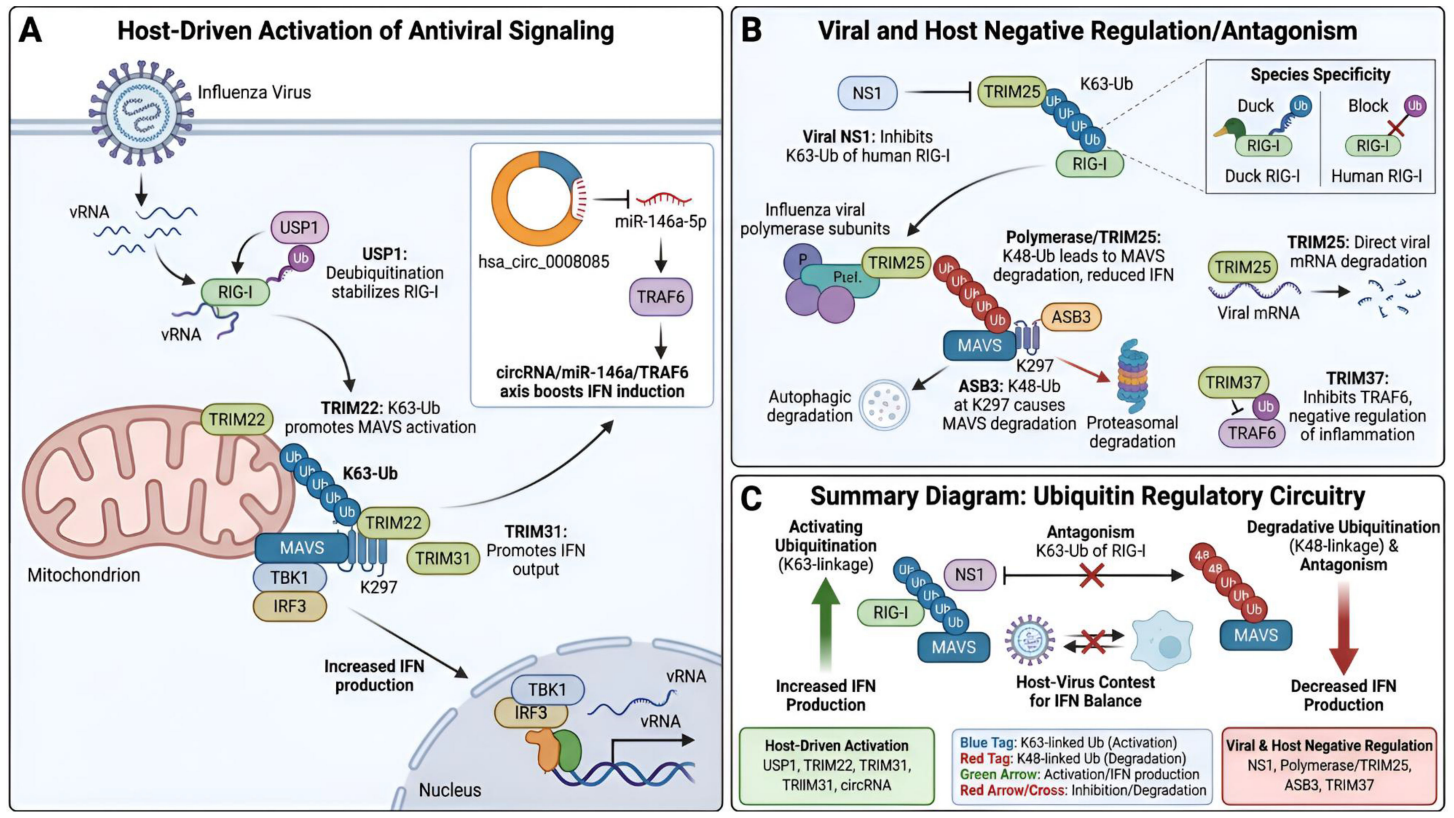
Ubiquitin-dependent regulation of the RIG-I–MAVS antiviral signaling pathway during influenza virus infection. **(A)** Host-driven activation involves K63-linked ubiquitination of MAVS by TRIM22 and TRIM31, USP1-mediated deubiquitination that stabilizes RIG-I, and circRNA hsa_circ_0008085-mediated enhancement of TRAF6 expression to promote TBK1/IRF3 recruitment and type I IFN production. **(B)** Viral antagonists, including NS1-mediated inhibition of TRIM25 and polymerase subunit–TRIM25-dependent K48-linked ubiquitination and degradation of MAVS, together with host negative regulators such as ASB3 and TRIM37, suppress antiviral signaling. **(C)** Summary of the ubiquitin regulatory circuitry showing the balance between activating K63-linked ubiquitination and degradative K48-linked ubiquitination in controlling interferon responses during influenza virus infection.

Collectively, the RIG-I–MAVS pathway is governed by a multilayered ubiquitin regulatory network in which activating ubiquitination events drive interferon production. In contrast, viral proteins and host negative regulators counterbalance these signals through degradative ubiquitination and pathway antagonism. This dynamic ubiquitin circuitry allows influenza viruses and host cells to reshape innate immune signaling, thereby influencing infection outcomes in a context-dependent manner (**Figure 5**).

#### 8.3.2 Viral antagonism of ubiquitin pathways

Influenza viruses use a range of mechanisms to evade the host’s ubiquitin-based antiviral response, either by interfering with ubiquitin-mediated degradation of antiviral response regulators or by exploiting host-cell ubiquitin receptors required for viral entry. These mechanisms allow the influenza virus to modify ubiquitin-based pathways that normally coordinate interferon and proteostasis-based antiviral responses. These counteracting mechanisms allow the virus to modulate ubiquitin signaling pathways, which normally coordinate interferon responses and proteostasis-associated antiviral responses. This mechanism is achieved by the virus manipulating the ubiquitin-dependent degradation of innate immune transcription factors. In the case of influenza infection, the host cell expresses the protein NMI, which binds to IRF7 and recruits TRIM21, leading to ubiquitination and proteasomal degradation of IRF7. The absence of NMI stabilizes IRF7, leading to increased type I interferon production and reduced viral replication, indicating that the virus manipulates the NMI-TRIM21 ubiquitin pathway to inhibit antiviral responses (152).

The influenza virus also inhibits host stress-response pathways mediated by the ubiquitin system, thereby facilitating the early stages of infection. HDAC6 is a cytoplasmic deacetylase that contains a zinc-finger domain and is responsible for mediating aggresome formation and stress granule formation, which have intrinsic antiviral activity. The influenza virus uses ubiquitin-binding stress-response pathways to facilitate capsid uncoating during the early stages of infection. Disruption of HDAC6 and its interaction with ubiquitin significantly impairs influenza virus infection, suggesting that the virus relies on ubiquitin-associated stress response pathways for successful replication (153). These results suggest that the influenza virus employs two strategies to evade host immune responses mediated by the ubiquitin system, and that influenza virus infection is significantly associated with ubiquitin system utilization.

### 8.4 Caspase-mediated PTMs controlling programmed cell death

#### 8.4.1 Apoptosis, necroptosis, and pyroptosis regulation

The cell death pathways activated by IAV infection, including apoptosis, necroptosis, and pyroptosis, are closely linked and often share common upstream signaling mechanisms. An important event in the activation of cell death pathways by IAV is the recognition of the viral genomic RNA by the cytosolic sensor protein ZBP1/DAI, which activates RIPK3-dependent cell signaling and the formation of RIPK1, FADD, and caspase-8 complexes, thereby leading to the activation of parallel apoptosis and necroptosis cell death pathways (154). The RIPK3-MLKL cell signaling pathway not only activates necroptotic cell membrane damage but also activates the inflammasome and the maturation of inflammatory cytokines, and caspase-8 activates inflammatory cell death signaling in the absence of necroptosis (155). In macrophages, activation of the NLRP3-caspase-1-gasdermin D signaling pathway triggers pyroptosis, a cell death pathway (137).

These apoptotic pathways are also highly regulated to facilitate viral replication. The activation of various caspases, such as caspase 2, 3, and 6, facilitates efficient viral replication in airway epithelial cells by promoting nucleoprotein trafficking and replication-related activities. The pro-apoptotic protein Bik, which activates caspase 3, also helps facilitate viral replication (156). The cleavage of various cellular structures by activated caspases, such as cortactin, facilitates viral replication by promoting efficient progeny virion release (157). In contrast, nuclear pore complex remodeling by activated caspases facilitates the efficient export of viral ribonucleoproteins (158). Influenza viruses also use activated apoptotic proteolysis to counter host antiviral activities by exploiting the cleavage of HDAC4 by activated caspase 3 and the activity of PA-X, which downregulates HDAC4 gene expression (159).

Significantly, inflammatory pyroptotic pathways play a critical role in disease severity. Type I interferon signaling can induce a shift of infected epithelial cells toward a pyroptotic cell death pathway under particular conditions (160), and the caspase-3 cleavage of gasdermin E (GSDME) is associated with epithelial cell pyroptosis and lung inflammation, whereas a deficiency of GSDME is associated with reduced tissue damage and increased survival of infected animals (138). In all these contexts, the pharmacological modulation of these caspase-dependent cell death pathways has been shown to reduce pathology associated with cell death and, under some conditions, viral replication (135, 136, 161). In conclusion, influenza infection of a cell triggers a coordinated cell death response involving multiple pathways, including apoptosis, necroptosis, and pyroptosis. In these cell death pathways, common upstream sensors activate multiple cell death mechanisms that can limit or exacerbate viral replication.

#### 8.4.2 PTM-dependent inflammasome activation and consequences for viral replication

Post-translational signaling pathways regulate inflammasome activation during IAV infection and also affect viral replication. The activation of the RAF/MEK/ERK kinase cascade leads to downstream AP-1 transcription factor activity, which primes pro-IL-1β transcription, a necessary event that triggers NLRP3 inflammasome activation following infection (119). Inhibition of the MEK signaling cascade or AP-1 activity reduces pro-IL-1β levels, indicating that this phosphorylation-dependent transcriptional regulation is a significant priming event that leads to the maturation of inflammatory cytokines following IAV infection (119).

Additionally, the inflammasome-related proteolytic pathways may also directly affect viral replication. The proteolytic cleavage of the viral M2 ion channel by caspases impairs the interaction between M2 and the autophagy protein LC3, thereby affecting M2 membrane targeting and, consequently, filament formation and virus production (139). The proteolytic modification of viral proteins by host proteases activated during inflammatory signaling may thus affect the structural activities of viral proteins that play critical roles in efficient virus production and release. Overall, phosphorylation-mediated transcriptional priming of inflammasome proteins and caspase-mediated proteolytic modification of viral proteins may establish a regulatory interface between inflammatory signaling and efficient viral replication. These studies thus demonstrate the critical role of PTM-mediated inflammasome activation in modulating host inflammatory responses and also affecting the production of infectious influenza virus particles.

## 9 PTM-dependent transcriptional control of inflammation and antiviral programs

### 9.1 NF-κB-centered circuits

IAV infection has been widely reported to activate NF-κB, a key signaling hub involved in innate immunity, inflammation, and viral pathogenesis, and various environmental, viral, and host factors regulate this activation. High humidity and temperature conditions have been associated with increased severity of IAV infection in some experimental and epidemiological settings by changing the composition of the intestinal flora, increasing intestinal permeability, and … with reported activation of NOD1/RIP2/NF-κB pathways and increased production of proinflammatory cytokines (IL-2, IL-6) and decrease IFN-γ and IL-17A production, affecting Th17/Treg balance and downregulating pIgR/sIgA/IgA (114). In CD8+ T cell memory, TCR-mediated IKK2/NF-κB signaling activated after memory establishment has been reported to enhance maintenance of lung-resident memory (TRM) cells by upregulating Bcl-2 and CD122 but negatively regulate TRM differentiation by excessive NF-κB activation, which counteracts TGF-β-induced CD69/CD103/Runx3/Eomes expression, without affecting recirculating memory (120).

Age-associated virulence of H7N9 has been linked to PB1-F2 aggregates that cause mitochondrial cristae collapse, complex V dysfunction, and reverse electron transfer-dependent oxidized mtDNA release, which activates the cGAS-STING-NF-κB pathway to increase IFN-β and chemokine production, especially in aged mice (115). Sorbicillinoid HSL-2 has been reported to exhibit antiviral activity against IAV, potentially through PPAR-γ activation, inhibition of NF-κB-mediated TNF-α, IL-6, and IL-1β overexpression, and reduced lung inflammation in mice (131). Diethylcarbamazine (DEC) decreases H1N1pdm09 titer in epithelial cells through maintenance of IFN-λ1, upregulation of IFN-β at 24 h, and modulation of IL-8 through the MYD88/NLRP3/NF-κB pathwaywithout altering IFN-λ1 (140). During IAV-Streptococcus pneumoniae co-infection, IL-17RA-NF-κB-driven inflammation disrupts TGF-β1-dependent epithelial tolerance, enhancing TRAF6/NF-κB activation, tight junction loss, and bacterial dissemination (121).

MicroRNA-4776, which is induced early in the bronchial epithelium, targets NFκBIB mRNA, thereby decreasing NFκBIB and increasing NF-κB, thereby enhancing the antiviral response (141). Artemisia extracts inhibit H1N1 through TLR4/MyD88/NF-κB pathways, resulting in decreased IL-1β/IL-6/TNF-α, and HA/NA targeting to inhibit viral entry/release. Another Artemisia extract targets NP to inhibit ROS-mediated mitochondrial apoptosis via AIF (132). IAV NS1 targets NF-κB, which otherwise regulates IFNL1, but instead targets exon/intron regions, thereby decreasing RNAPII/H3K27ac and type III IFN transcription (116). PA-X targets NF-κB p65, thereby inhibiting nuclear translocation and phosphorylation, leading to decreased TNF-α, Nos2, IL-6, IL-2, and IFN-β (117). Shu-Feng-Jie-Biao formula relieves IAV lung injury through NF-κB p65/ERK1/2 phosphorylation, thereby decreasing MIP-2/TNF-α/MCP-1/CCL5/ROS (124). Andrographolide targets NF-κB/JAK-STAT, thereby decreasing viral loads and cytokine levels in mice, and can be combined with another inhibitor, CL-385319, which targets IAV entry (134). Conversely, inducible GBP7 facilitates replication by suppressing NF-κB/JAK-STAT-mediated type I/III IFN and cytokine responses (110). Collectively, NF-κB circuits appear to integrate environmental cues, viral effectors, and host modulators that together influence the balance between protective immunity and immunopathology during IAV infection, with therapeutic targeting of its activation offering avenues to mitigate severity.

### 9.2 AP-1/cFos and STAT axis

IAV infection has been shown to activate AP-1/cFos and STAT signaling pathways by phosphorylation-dependent post-translational modifications (PTMs), influencing replication, innate immunity, and immunopathogenesis. The AP-1 subunit cFos is upregulated in IAV-infected A549 cells and has been associated with enhanced viral replication by promoting nuclear activity that downregulates IFN-β and apoptosis, thereby improving cell survival and late viral protein (NA, M2) synthesis (118). The molecular basis of cFos upregulation involves reduced NA mRNA synthesis and progeny titers following cFos knockdown, linking cFos with innate response modulation by IAV (118). Concurrently, AP-1 has been reported to promote NLRP3 inflammasome priming by ERK/c-Jun-mediated transcriptional upregulation of pro-IL-1β mRNA following H3N2 infection (119). Type I IFNs and MEK inhibition (U0126) decrease AP-1-dependent pro-IL-1β transcription, reducing IL-1β maturation and inflammasome activation (119).

STAT PTMs play a double-edged role. p-STAT1 has been associated with enhanced IAV replication, in part through effects on vRNA synthesis and potentiates the inflammatory response (109). Inhibition of p-STAT1 by fludarabine, through its incorporation into IAV RNA, has been shown to reduce viral titers, cytokine storms, and lung pathology in H5N1-infected mice, thereby increasing survival rates (109). Early phosphorylation of STAT2 appears to be mediated, at least in part, by RIG-I/MAVS signaling, independent of type I IFNs and JAKs. Early p-STAT2 is essential for ISG expression and for restricting IAV replication (31). MAPK12 and Syk kinases mediate the phosphorylation of STAT2. Inhibitors of these kinases suppress the early phosphorylation of STAT2 and subsequent IAV replication (31). In a dose-dependent manner, H1N1 infection induces the phosphorylation of STAT1 and STAT3, which is associated with neutrophil infiltration, M1 macrophage polarization, and ALI (111). Inhibition of these STATs exacerbates pathology in low-dose IAV infection. Therefore, balanced phosphorylation of these STATs is critical for protective immunity against IAV infection (111). On the other hand, β-Sitosterol suppresses IAV infection by disrupting the RIG-I/IFN/STAT signaling pathway. In a dose-dependent manner, β-Sitosterol suppresses p-STAT1/2, NF-κB/p38 MAPK, and cytokine production while protecting type I alveolar epithelial cells from apoptosis (126). Collectively, AP-1/cFos and STAT signaling axes represent PTM-regulated pathways that can influence the balance between proviral processes and antiviral immunity, with dysregulated phosphorylation driving pathology and offering therapeutic targets.

## 10 PTM cross-talk networks shaping influenza infection outcomes

During influenza virus infection, PTMs operate within interconnected signaling networks rather than as isolated events. The integration of phosphorylation, ubiquitination, SUMOylation, proteolytic cleavage, and metabolic signaling dynamically coordinates viral multiplication, innate immune responses, and host cellular remodeling, positioning PTM cross-talk as an important contributor to infection outcomes. Influenza viruses can extensively remodel these interconnected PTM circuits, thereby creating signaling conditions that may favor replication while also modulating host defensive pathways.

One well-characterized cross-talk module involves phosphorylation-dependent regulation of ubiquitin signaling pathways. The host kinase cascades activated during infection often regulate the recruitment of ubiquitin ligases and thereby promote cross-talk with ubiquitin signaling pathways. The assembly of PKCα–MEK1–ERK2 kinase complexes on viral ribonucleoproteins has been reported to regulate phosphorylation-dependent viral trafficking and thereby establishes a signaling complex that integrates other PTM signaling pathways (28). These observations are consistent with the infection-induced regulation of Cullin-4 E3 ligase interaction networks and the subsequent ubiquitination of the viral polymerase subunits, highlighting a potential role for the host kinase-regulated environment in ubiquitin-dependent replication (29, 36). Ubiquitin-dependent degradation of the metabolic regulator AMPK further supports the involvement of these interconnected PTM pathways in viral replication (61).

Cross-talk between ubiquitination and proteolytic cleavage pathways is another important aspect of innate immunity regulation. Caspase-8-induced cleavage of CYLD, which is a deubiquitinating enzyme, has been associated with enhanced ubiquitination of RIG-I and TAK1. These modifications have been linked to activation of antiviral signaling pathways. Together, these findings illustrate how ubiquitin signaling pathways can be reprogrammed through PTM-dependent mechanisms (33, 125). Other ubiquitin ligases and deubiquitases targeting MAVS, TRAF6, and RIG-I signaling pathways demonstrate how ubiquitin signaling pathways can be directly reprogrammed through PTMs (34, 146, 150).

Regulatory switches involving SUMOylation have been proposed as an additional PTM cross-talk mechanism that contributes to antiviral immunity and viral adaptation. Remodeling of the SUMOylation landscape during infection has been reported to derepress endogenous retroviral elements and activate interferon signaling (40). However, it has been shown that influenza viruses use SUMO-interacting motifs on viral proteins to recruit host SUMOylated cofactors, thereby enhancing viral polymerase activity and potentially contributing to cross-species adaptation (38, 69). SUMOylation of viral structural proteins, such as matrix protein M1, has also been implicated in supporting viral stability and intracellular trafficking, indicating cross-talk with other PTM-regulated viral replication events (68).

PTM cross-talk networks also contribute to the regulation of programmed cell death processes during infection. The apoptotic, necroptotic, and pyroptotic cell death processes have been linked through kinase-mediated phosphorylation events, caspase activation cascades, and ubiquitin-mediated adaptor protein complexes (41, 42, 155). TAK1 kinase-mediated phosphorylation cascades have been reported to regulate cell death through RIPK1-dependent signaling and modulate inflammatory processes, demonstrating the ability of individual PTM-mediated signaling nodes to integrate multiple cell defense processes (41). Host cell-mediated proteolytic processing of viral proteins, exemplified by caspase-mediated processing of M2, represents bidirectional PTM cross-talk between viral and cell defense processes, as cell defense processes modulate viral assembly and release (139).

Finally, PTM cross-talk also includes metabolic reprogramming events that facilitate the establishment of a favorable intracellular environment for viral replication. Activation of kinase-dependent signaling pathways, including TAK1/RORγ and JAK/STAT3, upregulates cholesterol biosynthesis via SREBP2-dependent transcription, which may create conditions favorable for viral entry and assembly (15, 30). In summary, these studies suggest that influenza virus infection can reprogram host signaling through the interplay of multiple PTMs. The integration of the phosphorylation cascade, ubiquitin signaling, SUMOylation, and proteolytic signaling pathways appears to be important for establishing a coordinated signaling environment that shapes stage-specific infection outcomes, highlighting PTM cross-talk as a significant contributor to influenza virus pathogenicity.

## 11 Translational implications

### 11.1 Host kinase inhibitors

Host-targeting strategies involving kinase-mediated PTMs represent promising therapeutic approaches for mitigating IAV infection by interfering with viral exploitation of cellular signaling while potentially reducing the likelihood of rapid drug resistance. Natural compounds and traditional Chinese medicine (TCM) derivatives have been reported to exhibit inhibitory activity on key kinases involved in inflammation, apoptosis, and viral replication. Theaflavin-3′-gallate (TF2b), an active compound derived from black tea, inhibits H1N1-induced inflammatory responses by downregulating TLR4/MAPK/p38 signaling, thereby reducing pro-inflammatory cytokines, including IL-6, TNF-α, and IL-1β, and chemokines such as CXCL2 and CCL3, with consequent attenuation of influenza-associated pneumonia (122). Separately, Miquelianin, a flavonoid compound, has been reported to inhibit H1N1 replication and MAPK activation, reducing IL-6 and IL-1β expression and attenuating lung injury *in vivo* (122). Together, these examples illustrate how modulation of MAPK-associated PTM signaling may help separate antiviral effects from excessive inflammatory pathology (122, 123). Yinchenhao Tang (YCHT) alleviates lung injury in H1N1-infected mice and has been linked to inhibition of JAK/STAT phosphorylation and reduced pro-inflammatory cytokine secretion. UPLC-Q-Orbitrap HRMS and network pharmacology identified 26 active serum compounds associated with these effects (128). Haoqin Qingdan Tang (HQQDT) reduces H1N1 viral loads and inflammation via JAK/STAT inhibition, downregulating IL-6/TNF-α/IL-1β/IFN-α/IFN-β, with HPLC identifying 12 active components and network analysis confirming STAT1/2/3 targeting (127).

The Shu-Feng-Jie-Biao formula reduces H1N1 lung damage by suppressing NF-κB p65/ERK1/2 phosphorylation, lowering MIP-2/TNF-α/MCP-1/CCL5/ROS, and reducing neutrophil-derived cytokines (124). Andrographolide suppresses H1N1 influenza in mice by inhibiting NF-κB/JAK-STAT signaling, lowering viral loads and cytokines, and collaborating with the entry inhibitor CL-385319 (134). β-Sitosterol inhibits RIG-I/IFN/STAT cross-talk, decreasing p-STAT1/2/NF-κB/p38 MAPK and proinflammatory cytokines (IL-6/TNF-α/IL-1β), and mitigating alveolar epithelial apoptosis by downregulating pro-apoptotic factors, thereby improving survival in infected mice (126). Selenium nanoparticles loaded with ribavirin (Se@RBV) suppress H1N1 infection by reducing cleaved PARP, cleaved caspase-8, and the pro-apoptotic Bax/Bak axis, while lowering p-p38, JNK, and p53 phosphorylation; these effects preserve mitochondrial integrity *in vitro* and reduce lung apoptosis *in vivo* (161). Baicalin inhibits H1N1-induced pyroptosis in alveolar epithelial cells by inhibiting caspase-3/GSDME activation and decreasing GSDME-N pore formation and lactate dehydrogenase release, as indicated by the reduction of bubble-like protrusions and lung tissue protection (135). 14-Deoxy-11,12-didehydroandrographolide (DAP) suppresses H5N1 apoptosis in A549 cells via caspase-9 inhibition, preventing mitochondrial cytochrome c release and Bax/Bcl-2 imbalance, without affecting caspase-8 (136). Collectively, these inhibitors largely target MAPK, JAK-STAT, and caspase signaling axes to curb IAV-induced PTM-driven inflammation and cell death, highlighting TCM-derived compounds as potential host-directed antiviral candidates for mitigating for mitigating pathogenesis.

### 11.2 Targeting PTM-modulating enzymes for antiviral strategies and vaccine design

Neddylation inhibition has emerged as a promising strategy for modulating IAV replication and inflammation in hosts. The NAE1 inhibitor MLN4924 blocks NEDD8 conjugation and disrupts activation of Cullin-RING ligases (CRLs) and NF-κB/IKK pathways. This intervention suppresses viral polymerase activity and viral titers in A549 cells and improves survival in IAV-infected mice (102). Glycosylation-guided vaccine design represents another strategy to address antigenic variation by exploiting PTMs. N-linked glycosylation (NLG) sites were introduced into the H7N9 HA to improve protein stability and immunogenicity by masking non-conserved epitopes and providing strong protection against homologous challenge in ferrets, which is superior to wild-type HA vaccines (83). A related challenge for glycan-aware and broadly protective vaccine design is how to present conserved or subdominant epitopes without inducing dominant off-target responses against the display scaffold itself. Romanov et al. recently showed that DNA origami-based virus-like particles displaying a germline-targeting HIV Env immunogen elicited no detectable scaffold-specific antibody responses (162). Compared with a state-of-the-art clinical protein nanoparticle, these DNA-VLPs increased the expansion of epitope-specific germinal-center B cells relative to off-target B cells and enhanced bnAb-lineage B cell expansion in a humanized mouse model of CD4-binding-site priming (162). Although this study was performed in the HIV vaccine setting rather than influenza, it establishes an important scaffold-design principle for next-generation viral vaccines: minimizing scaffold immunogenicity can help focus germinal-center competition toward rare B cell lineages recognizing conserved vaccine targets. This principle may be relevant to influenza bnAb vaccine development, particularly for multivalent display of glycan-aware HA immunogens designed to recruit B cells against conserved, subdominant, or conformationally constrained HA epitopes. Combining ubiquitin degradation and mRNA vaccine technology, the Ub-Re-NP mRNA vaccine induces Ub-Re-NP proteins to be degraded by the proteasome to reveal conserved epitopes and induce strong CD8+ T cell responses and IFN-γ production to provide cross-protection against H1N1 and influenza B in mice (163).

Host-targeting approaches may also modulate PTM enzymes, thereby enhancing antiviral activity. Artemisia extracts have been reported to bind HA and NA, thereby inhibiting viral entry and release. These extracts also suppress TLR4/MyD88/NF-κB activation and reduce inflammatory cytokine expression, leading to decreased lung inflammation and viral replication in mice (132). The sorbicillinoid compound HSL-2 activates PPAR-γ, thereby inhibiting NF-κB-mediated transcription of TNF-α, IL-6, and IL-1β, thereby inhibiting viral replication in A549 cells and alleviating pulmonary lesions in mice infected with the virus. The effect is reversed through PPAR-γ siRNA and inhibitor T0070907 (131). Diethylcarbamazine (DEC) decreases H1N1pdm09 titers in epithelial cells by maintaining IFN-λ1 expression, temporarily increasing IFN-β at 24 hours, and modifying IL-8 via MYD88/NLRP3/NF-κB pathways, while without affecting IFN-λ1 levels (140). Collectively, these approaches, ranging from neddylation/proteasome inhibition to glycosylation-optimized immunogens and PTM-targeted signaling modulation, may provide a basis for the development of host-directed antiviral agents and improved vaccines.

## 12 Future perspectives

Future advances in multi-omics research are likely to further clarify dynamic changes in the PTM landscape during IAV infection. Phosphoproteomic analysis of IAV entry into A549 cells revealed that virus-activated Cdc42-mediated signaling induces filopodia formation to facilitate endocytic uptake, which is negatively regulated by cortactin phosphorylation and translocation to the cortex to limit filopodia formation and uptake (54). Ubiquitination analysis of the IAV polymerase identified 59 lysine sites on the viral protein, which are regulated by K6-linked ubiquitination of PB1 at K578 to enable charge neutralization for the conformational transition required for PB2 N-terminal loop coordination in dimerization and vRNA replication (16). m5C-transcriptome-wide analysis of lncRNAs in H1N1-infected A549 cells revealed 1317 m5C peak sites that were upregulated and 1667 m5C peak sites that were downregulated, which were associated with protein modification and organelle localization, and 234 lncRNAs that regulated pathogen recognition and pathogenesis by differentially modified and expressed (97). Data-independent acquisition (DIA)-MS analysis was also used to improve quantitative analysis of glycosylation sites in IAV A/Switzerland/9715293/2013 (H3N2) variants, which was based on Tanimoto similarity to compare site-specific changes between WT/mutant and egg/cell culture-expressed proteins (84). Affinity purification-MS profiled CRL4 interactomes, identifying rewiring in IAV-infected cells, with DDB1/DCAF11/12L1 mediating non-degradative PB2 polyubiquitination for optimal polymerase activity (29).

Strain-specific PTMs play a vital role in antigenic evolution. Quantification of H1N1 antigenicity was performed using HA mutations and N-glycosylation variations via multi-task learning sparse group LASSO (MTL-SGL). It validated the antigenic site at positions 54, 125, and 160, which are close to the antibody-binding/receptor sites (82). Evolutionary studies on H3N2 IAV in southern China showed that HA1 160T blocks RBC binding. At the same time, 158–160 N-X-T enables glycosylation, which is essential for receptor interaction, as confirmed by C18 Chip-Q-TOF-MS (71). Mathematical modeling approaches have also been used to integrate PTMs with systems biology. An ODE model on innate immunity in IAV-infected mice was used to predict age-specific production rates of JAK-STAT activator (IFN/IL-6) using Bayesian/Monte Carlo methods. The results showed that higher production rates in juveniles are associated with increased pathology (112). Genome-wide CRISPR-based editing was used to screen IAV infection, showing that paxillin delta K68 K6 ubiquitination regulates IAV entry, with depletion resulting in increased replication through endosome modification (63). HA acetylation at K157/169/418/459 modulates pH stability and virulence; deacetylation mimics attenuate, while K418R yields safe, protective live-attenuated vaccines (18). Together, these advancements in mapping, identifying strain characteristics, modeling, and precise editing provide a more integrated view of the PTM network, informing the development of specific antivirals and vaccines to combat evolving IAV threats.

## 13 Conclusion

Collectively, available evidence supports the view that PTM signaling provides an additional systems-level regulatory layer of regulation that intersects with viral replication, immune modulation, host adaptation, and pathogenicity during influenza infection. Across phosphorylation, ubiquitination, SUMOylation, glycosylation, acetylation, lipidation, and epitranscriptomic RNA modifications, many reported mechanisms converge on polymerase function, ribonucleoprotein trafficking, virion assembly, and host metabolic remodeling. However, the strength and generalizability of evidence vary considerably across PTM types, individual modification sites, viral strains, and experimental models. Accordingly, while influenza viruses can exploit host PTM enzymes and signaling networks to support replication and immune evasion, it remains an important goal to define which PTM nodes are broadly conserved and rate-limiting *in vivo*. Framing influenza biology through the lens of PTM networks offers a conceptual framework that may link molecular regulation to viral evolution and support the development of host-directed antiviral and vaccine strategies. However, continued quantitative and physiologically relevant validation remains essential for prioritizing actionable targets.

Looking forward, a major challenge will be to define the spatiotemporal architecture of PTM networks across infection stages and host species, integrating quantitative PTM-omics, structural proteomics, and single-cell systems biology to resolve how combinatorial modification patterns control viral phenotypes. Such multi-omics mapping may help identify PTM network hubs that represent promising therapeutic nodes, particularly host enzymes whose inhibition could affect multiple viral processes while potentially limiting resistance development. In parallel, rational manipulation of PTM-dependent antigen glycosylation and ubiquitin-guided epitope presentation offers promising avenues for next-generation vaccine engineering that can induce broader and more durable protection. Ultimately, viewing influenza virus biology through the lens of integrated PTM systems provides a unifying conceptual framework that links molecular regulation to viral evolution and supports the continued development of host-directed antiviral and vaccine strategies to counter both seasonal and emerging pandemic strains.
